# Prognostic Value of Pretreatment Circulating Tumor HPV DNA Load in HPV-Associated Cancers: A Systematic Review and Meta-Analysis

**DOI:** 10.3390/ijms27104263

**Published:** 2026-05-11

**Authors:** Iwona Agnieszka Jabłońska, Marcin Goławski, Dorota Ścieglińska, Urszula Kacorzyk, Natalia Wojciuszkiewicz, Tomasz Wojciech Rutkowski, Agnieszka Maria Mazurek

**Affiliations:** 1 Clinical Trials Support Centre, Maria Sklodowska-Curie National Research Institute of Oncology Gliwice Branch, Wybrzeze Armii Krajowej 15, 44-102 Gliwice, Poland; 2 Center for Translational Research and Molecular Biology of Cancer, Maria Sklodowska-Curie National Research Institute of Oncology Gliwice Branch, Wybrzeze Armii Krajowej 15, 44-102 Gliwice, Poland; 3 Department of Pharmacology, Faculty of Medical Sciences in Zabrze, Medical University of Silesia, 41-808 Katowice, Poland; 4 Department of Biotechnology and Nutrigenomics, Institute of Genetics and Animal Biotechnology of the Polish Academy of Science, Postepu 36A St., Jastrzebiec, 05-552 Magdalenka, Poland; 5 I Radiation and Clinical Oncology Department, Maria Sklodowska-Curie National Research Institute of Oncology Gliwice Branch, Wybrzeze Armii Krajowej 15, 44-102 Gliwice, Poland

**Keywords:** ctHPV DNA, HPV-associated cancer, liquid biopsy, ctDNA, progression-free survival, overall survival, tumor burden

## Abstract

The qualitative detection of circulating tumor human papillomavirus DNA (ctHPV) has shown promise in HPV-associated cancers. We performed a systematic review and meta-analysis to evaluate the association of pretreatment ctHPV levels with survival outcomes and quantitative tumor burden metrics. Databases were searched through 5 January 2026. Studies were eligible if they included patients with HPV-associated cancers and reported quantitative pretreatment ctHPV levels in relation to survival outcomes or tumor burden. Twenty-three studies were included in the quantitative synthesis. Higher pretreatment ctHPV levels were associated with poorer progression-free survival (PFS) (8 studies, n = 883; HR = 1.86, 95% CI 1.19–2.90; *p* = 0.013). This association was primarily driven by studies in oropharyngeal cancer (OPC; HR = 2.47, 95% CI 1.59–3.84; *p* = 0.007), whereas no significant association was observed in cervical cancer. In multivariable analyses, elevated ctHPV remained associated with shorter PFS (HR = 1.87, 95% CI 1.66–2.10; *p* = 0.002). No significant association was observed for overall survival. Pretreatment ctHPV correlated with nodal volume in OPC (r = 0.45), nodal and tumor volume in OPC/anal cancer (r = 0.39), primary tumor volume in OPC (r = 0.22), and tumor diameter in cervical cancer (r = 0.44). Higher pretreatment ctHPV levels are associated with greater tumor burden and poorer PFS in HPV-associated OPC. CtHPV shows potential as a prognostic biomarker, although further prospective studies and assay standardization are needed.

## 1. Introduction

Infection with high-risk human papillomavirus (HPV) is a well-established etiologic factor in several malignancies, most notably cervical cancer (CC), oropharyngeal cancer (OPC), and anal cancer (AC). Globally, approximately 630,000 new HPV-associated cancers are diagnosed each year, accounting for 4.5% of all cancer cases diagnosed annually [1]. In light of the considerable global burden of HPV-associated cancers, there is an increasing need for sensitive and non-invasive tools to support early diagnosis, evaluation of treatment response, and surveillance.

One promising approach is the use of circulating cell-free DNA (cfDNA), which originates from apoptotic and necrotic tumor cells and can be detected in the plasma of cancer patients with various malignancies [2]. As cfDNA reflects tumor dynamics, it enables real-time assessment of disease status through liquid biopsy [3]. Circulating tumor DNA (ctDNA) is the tumor-derived fraction of cfDNA that is specific to cancer. One of its subtypes, circulating tumor HPV DNA (ctHPV), has attracted considerable interest in the context of HPV-associated cancers. This viral DNA, released into circulation from tumor cells, has demonstrated promise as a highly specific and sensitive biomarker for the presence of active disease [4,5].

Several studies have shown that detectable ctHPV following definitive therapy is strongly associated with residual disease and an increased risk of impending recurrence [6,7]. Furthermore, some studies suggest that the reappearance of ctHPV during post-treatment surveillance may serve as an early indicator of cancer recurrence [4,5]. However, the current literature, including meta-analyses, predominantly emphasizes qualitative detection (presence vs. absence) of ctHPV, while the prognostic implications of its quantitative levels, particularly before treatment, remain incompletely characterized. Recent findings suggest that baseline ctHPV levels, hereafter also referred to as viral load (VL) in line with terminology used across the original studies, may reflect tumor burden [8,9] and disease stage [10,11], which could have prognostic significance. Moreover, some studies demonstrate an association between pretreatment level of ctHPV and progression-free survival (PFS), disease-free survival (DFS), metastasis-free survival (MFS), or overall survival (OS) in patients with HPV-associated cancers [8,12,13,14,15,16]. To date, no meta-analysis has provided a quantitative synthesis of the available data regarding the prognostic value of pretreatment ctHPV levels in HPV-related cancers.

The aims of this meta-analysis are therefore twofold: (1) to quantitatively assess the association between pretreatment ctHPV levels and survival outcomes; and (2) to explore correlations between ctHPV levels and quantitative tumor burden metrics.

## 2. Methods

This meta-analysis was registered in the International Prospective Register of Systematic Reviews (PROSPERO; CRD42025627557) [17]. Minor amendments were introduced to the protocol after registration, limited to administrative details (funding information). The study was planned in accordance with the Preferred Reporting Items for Systematic Reviews and Meta-Analyses (PRISMA) and its extension [18]. The PRISMA 2020 checklist is provided in the Supplementary File S1.

### 2.1. Search Strategy

We searched PubMed, Scopus, Embase, and Web of Science on 20 July 2024, to identify eligible studies. No restrictions were applied regarding language or publication date at the search stage. An updated search using the same strategy was subsequently performed on 5 January 2026. The full database-specific search strings are provided in Supplementary File S2.

### 2.2. Eligibility Criteria

Our analysis included studies that met the following inclusion criteria: (i) patients who had a diagnosis of HPV-associated cancer; (ii) the article described a quantitative assessment of ctHPV levels in patients’ blood; and (iii) the article investigated an association between the ctHPV levels in blood and survival outcomes or a correlation between ctHPV levels and quantitative tumor burden metrics. Studies that did not report hazard ratios (HRs) and 95% CIs were included in the meta-analysis if these values could be reconstructed based on other reported data. Articles were excluded if (i) they were abstracts, reviews, letters, commentaries, case reports, case series, or study protocols; (ii) full-text articles were not available in English (for feasibility of data extraction); or (iii) multiple reports were based on the same patient population. When multiple reports were available for the same cohort, the most recent publication was included. Each report was assessed independently by two researchers, and disagreements were resolved by consensus or consultation with a third reviewer.

### 2.3. Data Extraction

The data were extracted from relevant articles by two independent researchers. Discrepancies were resolved through discussion with a third researcher. Extracted information included study characteristics, patient demographics, ctHPV measurement methods, and relevant clinical outcomes, which included correlation coefficients of ctHPV levels and various quantitative measures of tumor burden or HR with appropriate error estimates for PFS and OS. For 7 studies [13,14,19,20,21,22,23] presenting Kaplan–Meier survival curves stratified by ctHPV level (high vs. low based on median), survival estimates were extracted using WebPlotDigitizer (version 5.2) [24]. The plots were digitized by calibrating axes and manually extracting complete and censored observations from the high and low ctHPV groups. Contact with authors was attempted in cases where relevant data were missing or unclear.

Volumetric terminology was standardized, with metrics reported as gross primary tumor volume (GTV-T), gross nodal tumor volume (GTV-N), and total gross tumor volume (GTV-T+N). Study-specific labels (e.g., tumor volume, pGTV, nodal volume, nGTV) were considered synonymous with these categories when they referred to baseline gross disease. PET-derived metrics were likewise harmonized when the same definition was reported by at least 2 studies. Labels referring to metabolic tumor volume (MTV) were uniformly designated MTV50-N for nodal MTV at 50% of maximum standardized uptake value (SUVmax) and MTV50-T for primary tumor MTV at 50% SUVmax.

### 2.4. Statistical Analysis

All statistical analyses were conducted using the R programming environment (version 4.4.0, R Foundation for Statistical Computing, Vienna, Austria). Meta-analyses were performed with the “metafor” package (version 4.8-0), with data processing supported by “dplyr” (version 1.1.4) and Excel output managed using “openxlsx” (version 4.2.8).

Hazard ratios (HRs) and 95% confidence intervals (95% CIs), as well as correlation coefficients, were obtained directly from individual publications or calculated based on published survival curves or derived from available subgroup data, when sufficient data were available. Random-effects models with restricted maximum likelihood estimation (REML) were used to calculate pooled hazard ratios (HRs) and corresponding 95% confidence intervals (CIs). The Hartung–Knapp adjustment was applied to the random-effects model. For analysis, reported HRs were log-transformed. When only confidence intervals were available, the standard error (SE) was approximated using the formula [25]:

SE = [ln(Upper CI) − ln(Lower CI)] ÷ (2 × 1.96).(1)

Subgroup analyses for the association between ctHPV level and survival outcomes were predefined according to two criteria: (1) the clinical outcome assessed (OS, PFS, or DFS/PFS), and (2) tumor location (OPC, AC, or CC). Separate meta-analyses were performed only when at least three studies were available. Because too few studies evaluated ctHPV as a continuous variable, analyses according to the form of ctHPV assessment were not performed. OS and PFS were treated as the primary survival endpoints. Given the limited number of studies reporting DFS, it was not analyzed as a standalone endpoint but was considered only in an additional exploratory pooled analysis with PFS. The combined DFS/PFS analysis was considered exploratory and interpreted with caution.

Correlation coefficients were obtained directly from publications or computed from raw data. A random-effects model with REML and Hartung–Knapp adjustment was used to calculate pooled correlation estimates along with 95% CI. Fisher’s z transformation was utilized to normalize the distribution of correlation coefficients. The variance of z (Var z) was obtained by the following equation [26]:

Var z = 1 ÷ (number of patients − 3).(2)

Statistical heterogeneity was quantified using the tau2, H2, I^2^ statistic, and Cochran’s Q-test. Publication bias was assessed visually via funnel plots and statistically using Egger’s regression test. To evaluate the robustness of pooled results, leave-one-out sensitivity analyses were performed. Forest plots were generated to visualize the magnitude and precision of pooled HRs. Duval and Tweedie’s “Trim and Fill” method was applied to estimate a corrected effect size after adjusting for publication bias [27]. Moreover, a sensitivity analysis was performed stratifying studies by the source of hazard ratios (directly reported vs. reconstructed from Kaplan–Meier curves).

### 2.5. Risk of Bias

We used the Quality In Prognostic Studies (QUIPS) tool to assess the risk of bias in prognostic studies included in our analysis [28]. For observational studies evaluating associations (i.e., between ctHPV levels and tumor burden) without prognostic endpoints, we applied the National Institutes of Health (NIH) Quality Assessment Tool for Observational Cohort and Cross-Sectional Studies [29]. Each study was assessed by two independent researchers, and all discrepancies were resolved through discussion.

## 3. Results

### 3.1. Study Selection and Characteristics

**Figure 1:**
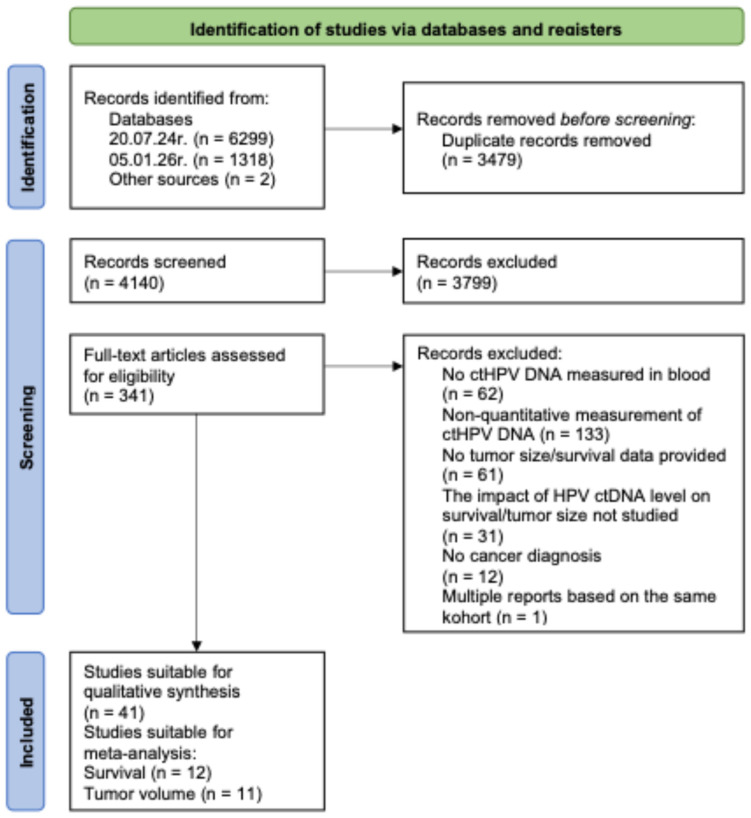
Flow chart of study search and selection according to PRISMA 2020 [18].

The literature search strategy retrieved 7617 records, and 2 studies were included from other sources. Two studies were identified through editorial comments that referred to eligible articles not captured by the initial search. After removing duplicates (n = 3479) and screening titles/abstract (n = 3799 records excluded), 341 full-text articles were assessed for eligibility. Of these, 300 studies were excluded at the full-text stage due to not meeting the inclusion criteria or for duplicating a report on a cohort from a more recent included study. As a result, we identified 41 articles eligible for qualitative synthesis; 23 reported comparable outcomes and were included in the meta-analysis (Figure 1). Detailed characteristics of the included studies are presented in Supplementary Table S1. Data on the association between pretreatment ctHPV level and patient survival outcomes were available for 18 studies, of which 12 were included in the meta-analysis. Associations with quantitative tumor burden metrics were explored in 27 studies; 11 provided data suitable for meta-analysis.

20 studies involved patients with head and neck cancers, with 15 specifically focusing on OPC [12,13,21,30,31,32,33,34,35,36,37,38,39,40,41]. Additionally, 4 studies examined only AC [8,9,16,42] and 15 included only patients with CC [10,15,19,20,22,23,32,43,44,45,46,47,48,49,50]. Two studies featured mixed-site cohorts [51,52]; however, in one of them [52], the correlation coefficient was provided only for the CC subgroup. Various analytical platforms were used across the studies, with droplet digital PCR (ddPCR) being the most common method (used in 30 studies) [8,9,10,14,15,16,20,21,22,23,31,32,33,34,36,37,39,40,41,43,44,45,46,47,50,51,52,53,54,55] including 7 studies that utilized the commercially available ctHPV assay NavDx (Naveris) [9,21,31,32,33,34,41]. Other techniques included quantitative PCR (qPCR, 9 studies) [12,13,30,35,38,48,49,51,52], digital PCR (dPCR, 1 study) [56], and next-generation sequencing (NGS, 5 studies) [19,22,42,56,57]. The amount of ctHPV was measured using various metrics, mostly copies/mL (28 studies) [8,10,12,13,14,15,22,23,30,33,35,36,37,38,39,40,43,44,47,48,49,50,51,52,53,54,55,56].

### 3.2. Association Between ctHPV Level and Survival Outcomes

Eighteen studies investigated the association between ctHPV levels and various survival outcomes: 10 assessed OS [12,13,14,15,16,20,22,23,43,58], 9 PFS [8,10,13,14,19,21,22,43,46], 2 DFS [15,16], 2 recurrence-free survival (RFS) [32,56], recurrence/persistence-free survival (RPFS) [35], MFS [12], locoregional recurrence-free survival (LRFS) [12], and disease-specific survival (DSS) [32].

Twelve of these studies, which involved a total of 1011 patients, contained data suitable for meta-analysis. Four articles focused on OPC (n = 468), 6 on CC (n = 441), and 2 on AC (n = 102). The association between pretreatment VL and OS, PFS, and DFS has been investigated in 9, 8, and 2 studies, respectively. Definitions of PFS and DFS varied slightly across the studies included. PFS was typically defined as the time from enrollment [19,22,43], treatment initiation [8], or diagnostic biopsy [14] to disease progression or death, with 2 studies referring to the standard PFS definition without further details [13,21]. Notably, in 2 studies, the definition of PFS did not include death as an event [19,22]. Moreover, for one study [12], PFS was calculated based on individual patient data, using treatment initiation as the starting point. DFS was defined either as the time from treatment initiation to recurrence or death [15] or, more broadly, also to include cases of lack of response to treatment [16]. Despite these differences, all endpoints shared the core objective of capturing time to treatment failure or disease recurrence.

The relationship between VL and survival outcomes was assessed continuously or by comparing outcomes between groups with high and low ctHPV levels. Cut-off values were determined using several approaches, including the median [13,14,15,16,21,22,23,43], mean [19], ROC curve analysis [8,12], or set arbitrarily [20]; they varied in the range of 33–3900 copies/mL. In two studies, ctHPV levels were expressed as a percentage of total cfDNA, with cut-off values ranging from 0.14 to 1.34% (Supplementary Table S1). Although HRs were available in the majority of the included studies, supplementary HRs for OS were calculated in 5 studies [13,14,20,22,23], and for PFS in 4 studies [13,19,21,22], using data extracted from Kaplan–Meier survival curves. The HRs included in our meta-analysis were primarily derived from univariate Cox proportional hazards models. However, HRs from multivariable analyses available in 3 studies [8,12,14] were analyzed separately as a subgroup.

#### 3.2.1. Association Between ctHPV and OS

**Figure 2:**
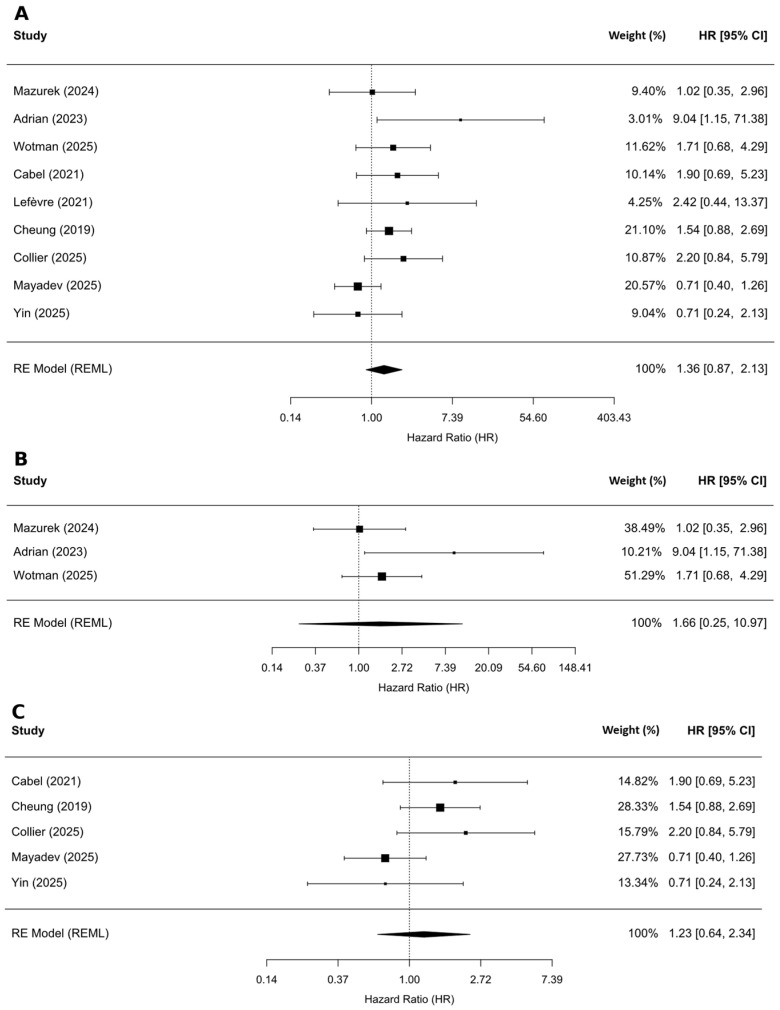
Associations between pretreatment ctHPV level and OS. (**A**) All cancer sites [12,13,14,15,16,20,22,23,43]; (**B**) oropharyngeal cancer subgroup [12,13,14]; (**C**) cervical cancer subgroup [16,20,22,23,43].

Nine studies including 892 patients assessed the association between OS and dichotomized ctHPV levels (high vs. low). Although higher ctHPV levels were associated with poorer OS, the pooled estimate was not statistically significant (HR = 1.36, 95% CI 0.87–2.13; I^2^ = 29.6%; *p* = 0.148; Figure 2A). When stratified by cancer site, the estimated effect was greater in OPC (HR = 1.66, 95% CI 0.25–10.97; *p* = 0.367) than in CC (HR = 1.22, 95% CI 0.64–2.34; *p* = 0.434; Figure 2B,C), although neither subgroup reached statistical significance.

#### 3.2.2. Association Between ctHPV and PFS or DFS

**Figure 3:**
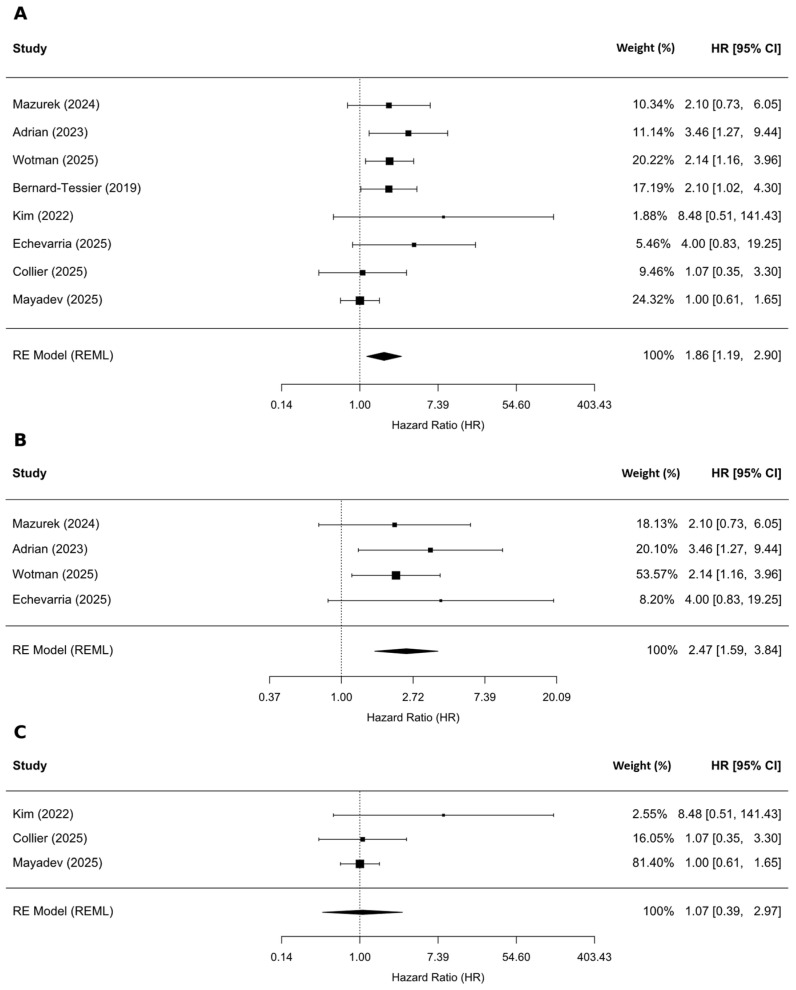
Associations between pretreatment ctHPV level and PFS. (**A**) All cancer sites [8,12,13,14,19,21,22,43]; (**B**) oropharyngeal cancer subgroup [12,13,14,21]; (**C**) cervical cancer subgroup [19,22,43].

Our analysis of 8 studies (n = 883) investigating the association between PFS and dichotomized pretreatment ctHPV levels showed an association between high VL and higher risk of recurrence (HR = 1.86, 95% CI: 1.19–2.90; *p* = 0.013), with low to moderate heterogeneity (I^2^ = 34.49%; Figure 3A). When we restricted analysis to the 4 studies involving patients with OPC (n = 468), the pooled HR was 2.47 (95% CI: 1.59–3.84; *p* = 0.007; Figure 3B). Conversely, in the subgroup restricted to studies including patients with CC, the pooled HR was 1.07 (95% CI 0.39–2.97; *p* = 0.802; Figure 3C). Across these subgroups, heterogeneity was minimal (I^2^ = 0–0.01%). Analysis of PFS among AC patients was not performed because there was only one study in this group.

**Figure 4:**
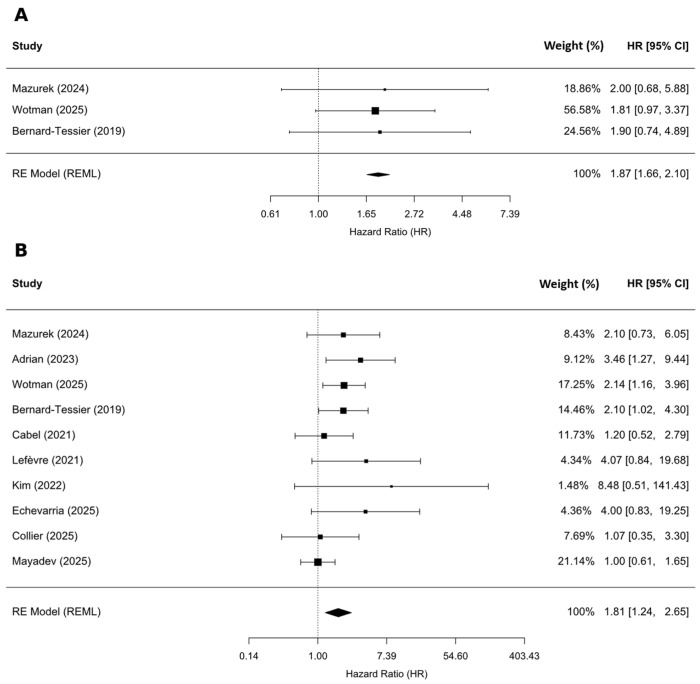
(**A**) Association between pretreatment ctHPV level and PFS derived from multivariate analyses (only OPC and AC patients) [8,12,14]; (**B**) Association between pretreatment ctHPV level and PFS/DFS across all cancer sites [8,12,13,14,15,16,19,21,22,43].

Regarding the association between pretreatment ctHPV levels and PFS, 3 studies reported HRs derived from multivariable Cox regression models. The pooled analysis of these studies demonstrated that elevated pretreatment ctHPV levels were significantly associated with shorter PFS (HR = 1.87; 95% CI: 1.66–2.10; *p* = 0.002) (Figure 4A), with no observed heterogeneity (I^2^ = 0%). This result indicates a consistent prognostic effect across studies that controlled for potential confounding variables.

Additionally, we analyzed the association between pretreatment ctHPV level and PFS and DFS jointly. In the pooled analysis of 10 studies, including 983 patients across OPC, AC, and CC, higher pretreatment ctHPV levels were significantly associated with shorter PFS/DFS (HR = 1.81; 95% CI: 1.24–2.65; *p* = 0.006; Figure 4B).

#### 3.2.3. Sensitivity Analyses and Risk-of-Bias Assessment in Survival Analysis

The QUIPS assessment of prognostic studies included in our analysis indicated that none of them was rated high in terms of risk of bias in any of the six domains (Supplementary Table S2).

Visual inspection of funnel plots (Supplementary Figures S1–S8) revealed asymmetry in some of the subgroups (Supplementary Figures S1, S2, S6 and S8); however, Egger’s test did not indicate significant publication bias in these analyses. We used a trim-and-fill method to adjust for publication bias under the random-effects model with Hartung and Knapp adjustment, and the results are shown in Supplementary Table S4, and Supplementary Figures S13–S18. Among the outcomes that were statistically significant in the primary analysis, namely PFS among all locations (Supplementary Figure S16), PFS in OPC (Supplementary Figure S17), DFS/PFS (Supplementary Figure S20), and the trim and fill method led to the imputation of 2, 1, and 3 studies, respectively. After adjustment, the pooled HRs for PFS across all cancer sites and for PFS in the OPC subgroup were 1.72 (95% CI 1.13–2.61; *p* = 0.01) and 2.37 (95% CI 1.62–3.47; *p* = 0.003), respectively. In the DFS/PFS subgroup, the pooled HR was 1.63 (95% CI 1.13–2.34), and the association remained statistically significant (*p* = 0.01). Moreover, using the trim and fill method, two studies were imputed in the subgroup of the multivariate HR for PFS (Supplementary Figure S19). In this case, pooled HR was 1.81 (95% CI 1.67–1.96), and the *p*-value was smaller than before adjustment (*p* < 0.001).

Sensitivity analyses using a leave-one-out approach demonstrated that the observed effect sizes were not significantly influenced by the exclusion of any single study, except for the OS analysis across all cancer sites (Supplementary Table S5). In this subgroup, exclusion of one study [43] strengthened the association and rendered it statistically significant (HR = 1.59, 95% CI 1.08–2.34; *p* = 0.025), while heterogeneity was reduced to nearly zero. This suggests that this study had a noticeable attenuating influence on the pooled OS estimate.

Additionally, a sensitivity analysis restricted to directly reported hazard ratios showed consistent results, with the main associations remaining statistically significant (Supplementary Table S8).

### 3.3. Association Between ctHPV Level and Quantitative Tumor Burden Metrics

The correlation between quantitative measures of disease burden and ctHPV levels was investigated in 28 studies. All studies included in the quantitative synthesis examined the correlation between disease burden and pretreatment ctHPV levels. Tumor burden was assessed using a wide range of metrics, primarily derived from radiologic imaging. One study used postoperative measurements [41]. Eleven studies, reporting Spearman correlation coefficients or providing individual patient-level data that allowed their calculation, were included in the meta-analysis, comprising a total of 603 patients [31,37,38,39,44,48,49,51,52,54,57]. Four distinct disease burden metrics were consistently reported and subjected to quantitative synthesis: GTV-T, GTV-N, GTV-T+N, and tumor diameter.

#### 3.3.1. Tumor Volume-Based Metrics

**Figure 5:**
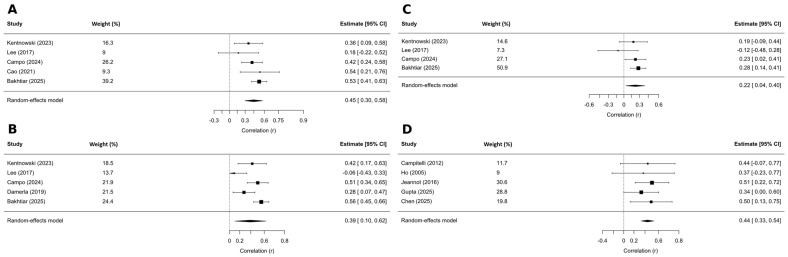
Correlations between pretreatment ctHPV level and quantitative tumor-burden metrics. (**A**) GTV-N in OPC [31,37,38,39,57]. (**B**) GTV-T+N in OPC + AC [31,38,39,51,57]. (**C**) GTV-T in OPC [31,38,39,57]. (**D**) Primary-tumor diameter in CC [44,48,49,52,54].

GTV-N was analyzed in 5 studies [31,37,38,39,57], which included a total of 368 patients with OPC, resulting in a pooled correlation of r = 0.45 (95% CI: 0.30–0.58, *p* = 0.002), with low heterogeneity observed (I^2^ = 21.7%). Five studies [31,38,39,51,57], including a total of 416 patients with OPC and 8 patients with AC, assessed the correlation between ctHPV levels and GTV-T+N. The pooled correlation coefficient was r = 0.39 (95% CI: 0.10–0.62, *p* = 0.02), with high heterogeneity (I^2^ = 74.8%); see Figure 5A,B.

A weaker, but still significant, association was observed between ctHPV levels and GTV-T, based on 4 studies [31,38,39,57] that included 340 patients with OPC. The pooled correlation coefficient was r = 0.22 (95% CI: 0.03–0.40, *p* = 0.03), with negligible heterogeneity (I^2^ = 0.2%; Figure 5C).

#### 3.3.2. Tumor Diameter-Based Metrics

Five studies [44,48,49,52,54], which enrolled a total of 126 patients with CC, analyzed the correlation between tumor diameters and ctHPV levels. There was a statistically significant pooled correlation between these variables (r = 0.44, 95% CI: 0.33–0.54, *p* < 0.001), with no evidence of heterogeneity (I^2^ = 0%; Figure 5D).

#### 3.3.3. Sensitivity Analyses and Risk-of-Bias Assessment in Tumor Burden Analysis

The quality of the included studies was evaluated using the NIH Quality Assessment Tool for Observational Cohort and Cross-Sectional Studies (Supplementary Table S3). Nine studies were rated as good, one as poor, and the remaining studies were judged to be of fair quality.

Visual inspection of funnel plots revealed no clear asymmetry across analyses, with the exception of the GTV-T subgroup in OPC (Supplementary Figures S9–S12). Nevertheless, Egger’s test was non-significant for all analyses (*p* > 0.05). Trim-and-fill analysis for the GTV-T subgroup in OPC imputed two potentially missing studies. After adjustment, the pooled correlation coefficient remained statistically significant at 0.26 (*p* < 0.001; Supplementary Table S6; Supplementary Figures S21–S24).

Leave-one-out analyses demonstrated that the pooled correlation estimates for tumor diameter in CC and nodal volume in OPC were robust to the exclusion of individual studies. In contrast, the pooled estimates for subgroups of GTV-T in OPC and GTV-T+N in OPC/AC were sensitive to the omission of single studies and remained statistically significant only after the exclusion of Lee et al. [57] (Supplementary Table S7). This suggests that Lee et al. may have attenuated the pooled estimates; however, given the sensitivity to a single study, these findings should be interpreted with caution.

## 4. Discussion

### 4.1. Baseline ctHPV Level and Survival Outcomes

Our systematic review and meta-analysis support the prognostic value of pretreatment ctHPV levels. Across HPV-associated cancers, elevated pretreatment ctHPV levels were significantly associated with poorer PFS. The pooled estimate indicated an 81% higher risk of progression in patients with high baseline ctHPV levels (HR = 1.81; *p* = 0.006). However, this overall association appeared to be largely driven by the OPC subgroup, where the strongest prognostic signal was observed (HR = 2.47; *p* = 0.007), whereas no clear association was identified in CC. Higher baseline ctHPV levels also remained significantly associated with shorter PFS in multivariable models adjusted for clinical covariates (HR = 1.87; *p* = 0.002). No statistically significant association was observed for OS (HR = 1.36; *p* = 0.148). Heterogeneity across analyses was low to moderate. The observed association of ctHPV level with PFS but not with OS may reflect several factors. In this context, pretreatment ctHPV load appears more closely related to disease dynamics than to overall mortality. OS is influenced by a broad range of variables, including post-progression treatments, comorbidities, or non-cancer-related mortality. Furthermore, limited follow-up duration and a low number of death events in HPV-associated OPC may reduce statistical power for OS analyses.

Several meta-analyses have addressed the utility of detecting ctHPV DNA, both in terms of its prognostic relevance and its role in post-treatment surveillance [59,60]. However, they did not specifically focus on the prognostic significance of VL levels. Chennareddy et al. [61] explored the prognostic relevance of pretreatment ctHPV levels in patients with HPV related OPC in a recent systematic review. The authors highlighted inconsistencies in the existing literature, referencing studies by Adrian et al. [13], Cao et al. [37], and Dahlstrom et al. [62]. Significant differences in study design and analytical approaches limit the ability to directly compare outcomes of these studies. Therefore, in our analysis, which specifically focused on this issue, we applied more rigorous inclusion criteria to enhance the comparability of the included studies.

The predictive value of ctHPV detection was beyond the scope of this meta-analysis, but this aspect remains of clear clinical interest. HPV-related OPCs have a considerably more favorable prognosis than HPV-unrelated cases, and potential treatment de-escalation is under investigation in clinical trials [63]. However, the current recommendation remains the same for both groups of patients. In this context, quantitative ctHPV measures—baseline levels and on-treatment clearance kinetics—may provide a biologically grounded basis for stratification. For example, Chera et al. used pretreatment ctHPV to identify a profile of its clearance kinetics to predict the likelihood of disease control during chemoradiotherapy [64], while Boyle et al. used pretreatment ctHPV to assess the risk of residual disease in patients undergoing curative surgery [65].

### 4.2. CtHPV Level as a Biomarker of Tumor Burden

To our knowledge, no prior meta-analysis has quantitatively synthesized the correlations between pretreatment ctHPV DNA levels and tumor volume metrics. We observed a moderate association between pretreatment ctHPV levels and several quantitative indicators of disease burden. Consistent associations were identified for GTV-N (r = 0.45), whereas the correlations with GTV-T+N (r = 0.39) and GTV-T alone (r = 0.22) were weaker, although still statistically significant. The correlation between pretreatment ctHPV levels and GTV-T+N showed substantial between-study heterogeneity (I^2^ = 74.8%). This heterogeneity may reflect differences in ctHPV detection platforms, HPV genotype coverage, imaging and segmentation protocols, and disease stage distribution. Furthermore, leave-one-out analyses for the GTV-T and GTV-T+N groups suggested that the study by Lee et al. attenuated the pooled estimates, as statistical significance was reached only after its exclusion. Therefore, these findings should be interpreted with caution. These results suggest that in patients with OPC, ctHPV levels may reflect overall disease burden, particularly nodal involvement, more accurately than primary tumor volume alone, and that lymph node metastases may contribute substantially to ctHPV release. This interpretation is consistent with the clinical presentation of HPV-related OPC, which is often characterized by bulky nodal disease and a relatively small primary tumor. Moreover, most patients with HPV-related OPC present with nodal metastases at diagnosis, while the primary tumor, which is most commonly located in the tonsil, may be undetectable at diagnosis because of prior diagnostic tonsillectomy [38,66,67,68,69].

As mentioned above, leave-one-out analyses in this study showed that the correlation with nodal volume was more robust than that observed for GTV-T and GTV-T+N. This pattern further supports the interpretation that pretreatment ctHPV DNA levels in OPC may be more closely linked to nodal disease burden than to primary tumor volume alone.

Associations between T stage or N stage and ctHPV levels were not evaluated in our meta-analysis; however, results reported in several studies appear consistent with our findings. Kentnowski et al. showed significantly higher ctHPV levels in N3 compared with N1, with no association between ctHPV and T stage [38]. Cao et al. likewise found no correlation between T stage and ctHPV levels [30]. Chera et al. showed that patients with T3-T4 stage HPV-related OPC had significantly lower pretreatment ctHPV level than patients with T2 disease [64]. The lack of correlation between T stage and ctHPV may reflect the fact that T classification captures the extent of infiltration and involvement of adjacent structures more than tumor volume.

Interestingly, in the CC subgroup, the pooled correlation of primary tumor diameter with ctHPV levels was r = 0.44, with *p* < 0.001. This suggests that the relationship between tumor extent and viral DNA release may differ across anatomical sites, tumor location (primary tumor site, nodal metastases), and the burden measure applied. In our dataset, all CC studies reported correlations with tumor diameter, whereas all OPC studies reported volumetric tumor measures. Therefore, the apparent site-specific difference may at least partly reflect differences in the reported imaging-derived metrics rather than a purely biological distinction.

Nevertheless, our results support the potential utility of ctHPV as a quantitative biomarker of tumor burden across HPV-related cancers and highlight the potential influence of tumor location and regional spread on ctHPV detectability in circulation.

### 4.3. Underlying Biological Mechanisms for ctHPV DNA as a Biomarker

HPV-related malignancies are characterized by integration of the viral genome into host cells and expression of viral oncoproteins, which drive malignant transformation and proliferation [70]. Circulating tumor-derived DNA can enter the bloodstream via apoptosis, necrosis, or active secretion from cancer cells [2,71]. Therefore, the amount of ctHPV detected in plasma or serum can reflect the overall tumor burden, including cancer cells that have spread to distant sites. One of the interesting findings in our study is that ctHPV levels exhibit stronger correlations with GTV-N or GTV-T+N than with GTV-T in patients with OPC. The reticulated crypt epithelium is closely associated with underlying lymphoid tissue and lacks a dense connective tissue barrier separating the epithelial compartment from the lymphoid microenvironment. This anatomical organization may partly explain lymph node involvement. Before the primary lesion grows enough to be detected by clinical or imaging examination, cancer cells from the basal cell layer migrate to the regional lymph nodes. In this way, metastatic lymph nodes grow simultaneously with the primary lesion or even sooner than the tumor in the primary site [72]. Compared to normal, metastatic lymph nodes are usually better vascularized and contain necrotic areas [73], which facilitates increased release of viral DNA fragments into the circulation.

At the same time, ctHPV shedding is likely multifactorial and may also be influenced by local biological factors, including chronic or acute inflammation within the oropharyngeal mucosa. Because OPC includes multiple anatomical subsites with potentially distinct biological behavior, this mechanism should not be interpreted as specific to all oropharyngeal tumors. Taken together, these observations support the hypothesis that ctHPV level may reflect not only tumor volume but also patterns of nodal dissemination and underlying tumor biology.

The observed association between pretreatment ctHPV and progression-free survival may be driven, at least in part, by the relationship between ctHPV level and disease burden. However, the persistence of this association in multivariable models suggests that ctHPV may provide prognostic information beyond tumor burden alone. This signal appeared strongest in OPC, which was also the only cancer type represented in the multivariable analyses. These findings support the view that pretreatment ctHPV in OPC may reflect both disease extent and additional biological features relevant to the risk of progression that are not fully captured by conventional clinicopathological factors.

### 4.4. Future Perspectives

Quantitative assessment of ctHPV may have clinical potential, as pretreatment viral load appears to carry prognostic information. This biomarker could potentially contribute to risk stratification, although its role in treatment decision-making remains to be established. Future studies should aim to standardize ctHPV quantification protocols, including harmonized definitions of VL units and thresholds. Multicenter prospective cohorts using validated ctHPV detection assays are still needed to confirm the prognostic utility of ctHPV in real-world settings. It should also be noted that the lack of standardization even in ddPCR-based assays (e.g., commercial NavDx vs. in-house ddPCR) prevents the establishment of a universal cutoff, which limits immediate clinical applicability.

Although the present meta-analysis could not formally compare analytical platforms, the most commonly used in biomarker ctHPV studies are discussed below. Droplet digital PCR is currently the most frequently represented method in the available prognostic ctHPV literature and offers several practical advantages for quantitative monitoring, including generally higher analytical precision than standard qPCR [61]. NGS-based approaches may provide even higher analytical sensitivity for ctHPV detection [74] and offer broader molecular characterization, including HPV genotyping and assessment of viral integration site. However, their use in prognostic settings remains limited, possibly owing to higher costs, increased technical complexity, as well as the need for more standardized bioinformatic pipelines. Future prospective studies should therefore directly compare analytical platforms using harmonized pre-analytical workflows, with standardized reporting units, positivity thresholds, and defined endpoints.

In addition, emerging evidence suggests that changes in ctHPV levels during or after treatment may also have prognostic value [9,13,75,76]. Therefore, the integration of serial quantitative ctHPV assessments and modeling of viral kinetics represents a promising direction for future trials.

### 4.5. Study Limitations

Several limitations of our study must be considered. Although 23 studies were included in the quantitative synthesis, the number of studies per some analytical subgroups was limited. In addition, considerable methodological heterogeneity was observed across studies, including variability in ctHPV quantification units (copies/mL, percentages) and in the definition of dichotomous thresholds (medians, means, or ROC-derived cut-offs). This lack of standardization may have introduced additional between-study variability and limited the comparability of pooled estimates.

Although most included studies were prospective, the observational nature of the data precludes strong causal inferences. Additionally, only a minority of studies reported multivariable-adjusted hazard ratios, limiting our ability to control for confounding. Our analyses relied on aggregate data, not individual patient data. Finally, publication bias also remains a concern, especially in analyses with fewer studies.

## 5. Conclusions

Current evidence suggests that higher pretreatment ctHPV levels are associated with greater tumor burden in HPV-related cancers and with poorer progression-free survival in patients with HPV-related oropharyngeal cancer, but not cervical cancer. These findings support the potential of ctHPV as a prognostic biomarker, although further prospective research is required to clarify its clinical utility. Standardization of quantification methods and clinically relevant cut-offs will be essential for future implementation.

## Data Availability

No new data were created or analyzed in this study. Data sharing is not applicable.

## Supplementary Materials

The following supporting information can be downloaded at: https://www.mdpi.com/article/10.3390/ijms27104263/s1. Reference [77] is cited in the Supplementary Materials.

## Abbreviations

The following abbreviations are used in this manuscript:

AC	anal cancer
AJCC	American Joint Committee on Cancer
CC	cervical cancer
cfDNA	circulating cell-free DNA
CHT	chemotherapy
CIs	confidence intervals
CRT	chemoradiotherapy
ctDNA	circulating tumor DNA
ctHPV	circulating tumor HPV DNA
ddPCR	droplet digital polymerase chain reaction
DFS	disease-free survival
dPCR	digital polymerase chain reaction
FFP	freedom from progression
FIGO	International Federation of Gynecology and Obstetrics
GTV-N	gross nodal tumor volume
GTV-T	gross primary tumor volume
GTV-T+N	total gross tumor volume
HNSCC	head and neck squamous cell carcinoma
HPV	human papillomavirus
HR	hazard ratio
LRFS	locoregional recurrence-free survival
MFS	metastasis-free survival
MTV	metabolic tumor volume
MTV50-N	nodal MTV at 50% SUVmax
MTV50-T	primary-tumor MTV at 50% SUVmax
MTV50-T+N	primary + nodal MTV at 50% SUVmax
NGS	next-generation sequencing
NIH	National Institutes of Health
OPC	oropharyngeal cancer
OS	overall survival
PFS	progression-free survival
qPCR	quantitative real-time PCR
QUIPS	Quality In Prognosis Studies tool
RECIST	Response Evaluation Criteria in Solid Tumors
REML	restricted maximum likelihood
RFS	recurrence-free survival
ROC	receiver operating characteristic
RPFS	recurrence/persistence-free survival
RT	radiotherapy
SCCUP	squamous cell carcinoma of unknown primary
SE	standard error
SUVmax	maximum standardized uptake value
TTB	total tumor burden
UICC	Union for International Cancer Control
Var z	variance of z
VL	viral load (ctHPV DNA level in plasma)
