# Supplementary material for: Prognostic Value of Pretreatment Circulating Tumor HPV DNA Load in HPV-Associated Cancers: A Systematic Review and Meta-Analysis
Source: Int J Mol Sci. 2026 May 11;27(10):4263. doi: 10.3390/ijms27104263 (PMC13207696; doi:10.3390/ijms27104263)
Supplement: Supplementary file 1 [file ijms-27-04263-s001.zip › Supplementary Figures S1-S24.pdf]

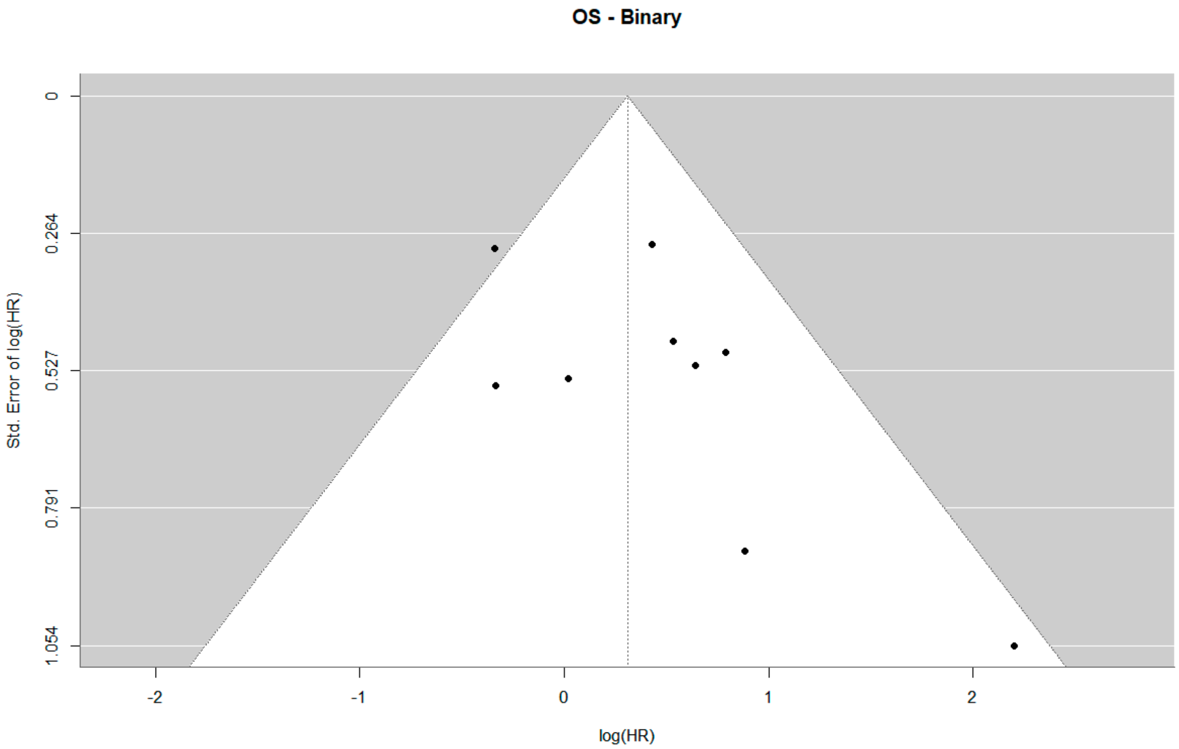

Supplementary Figure S1. Funnel plot for the association between pretreatment ctHPV (high vs low) and OS.

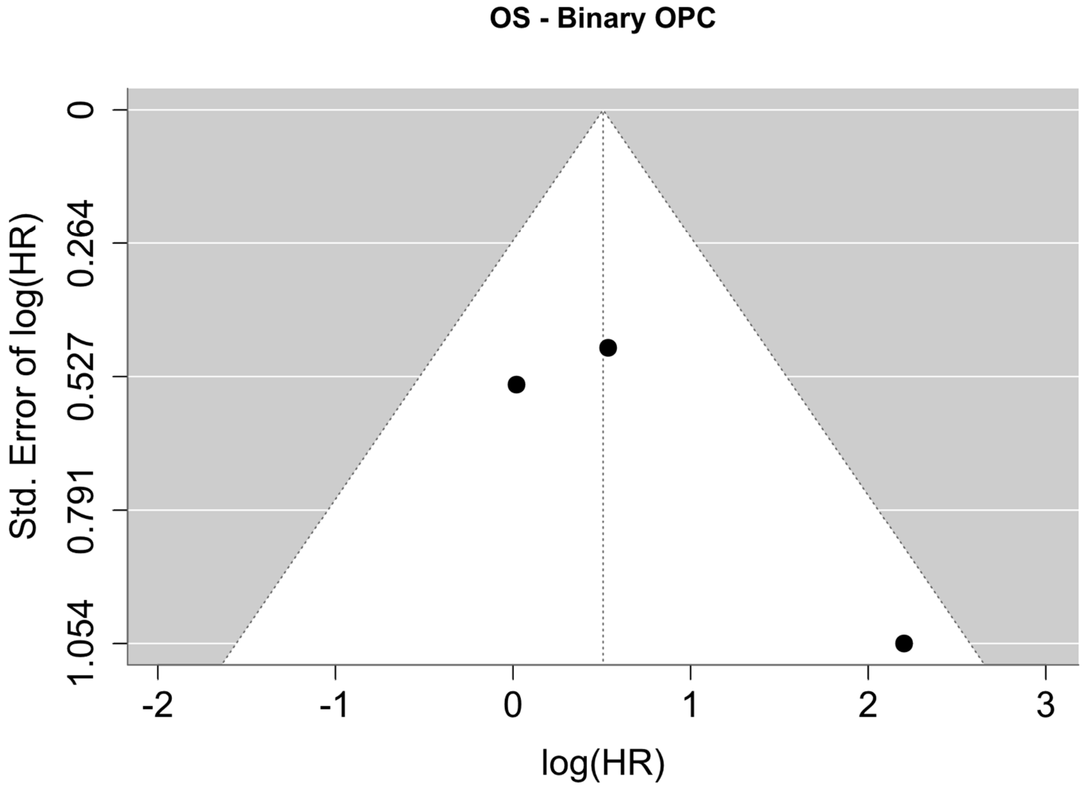

Supplementary Figure S2. Funnel plot for the association between pretreatment ctHPV (high vs low) and OS in the OPC subgroup.

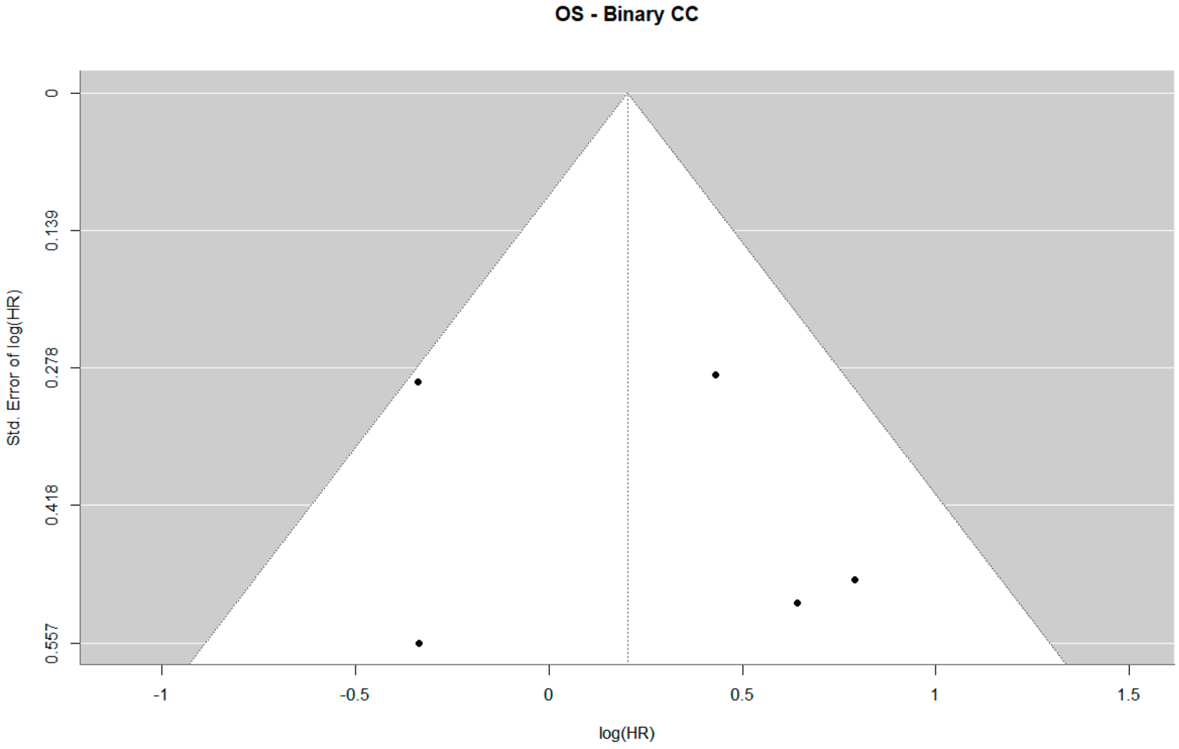

Supplementary Figure S3. Funnel plot for the association between pretreatment ctHPV (high vs low) and OS in the CC subgroup.

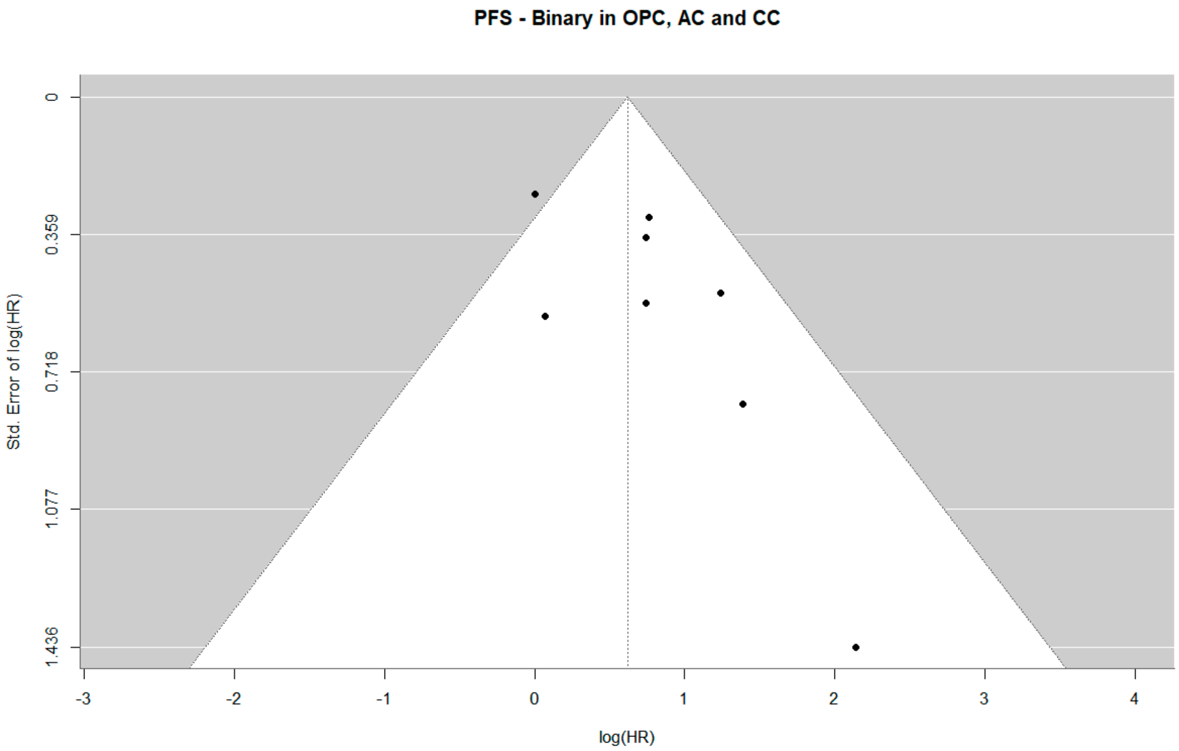

Supplementary Figure S4. Funnel plot for the association between pretreatment ctHPV (high vs low) and PFS (OPC, AC and CC).

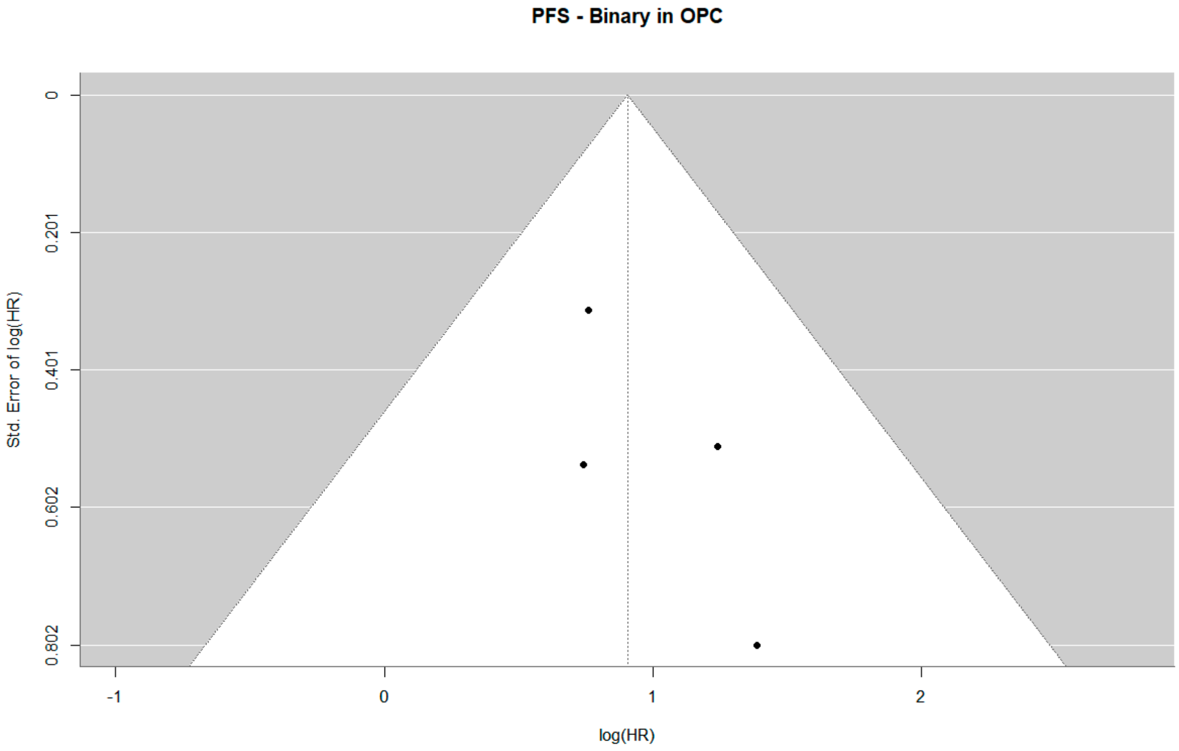

Supplementary Figure S5. Funnel plot for the association between pretreatment ctHPV (high vs low) and PFS in OPC subgroup.

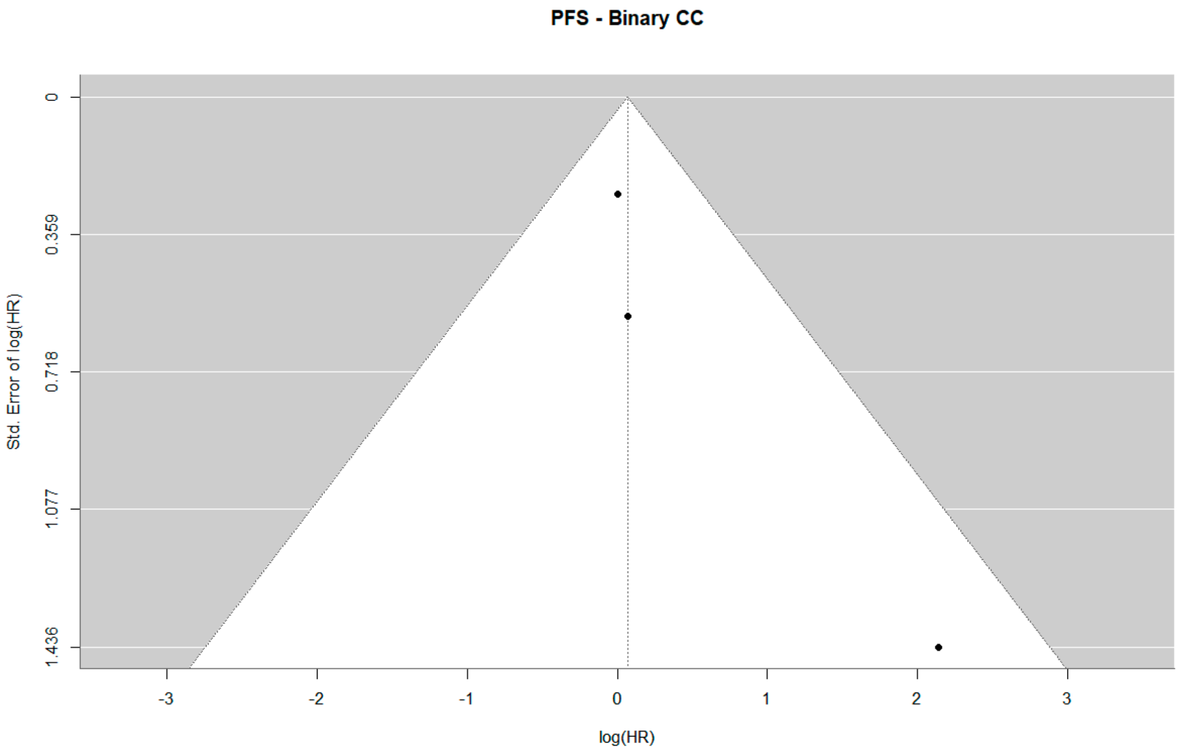

Supplementary Figure S6. Funnel plot for the association between pretreatment ctHPV (high vs low) and PFS in CC subgroup.

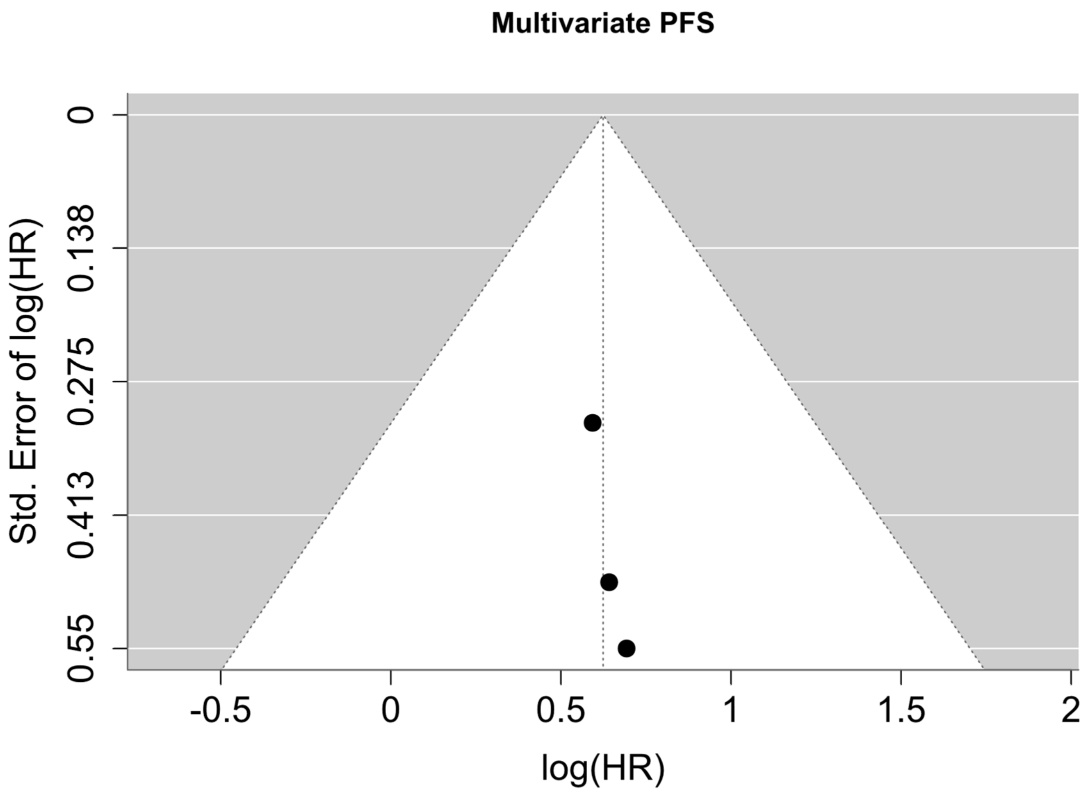

Supplementary Figure S7. Funnel plot for the association between pretreatment ctHPV (high vs low) and PFS in OPC subgroup – multivariate analyses.

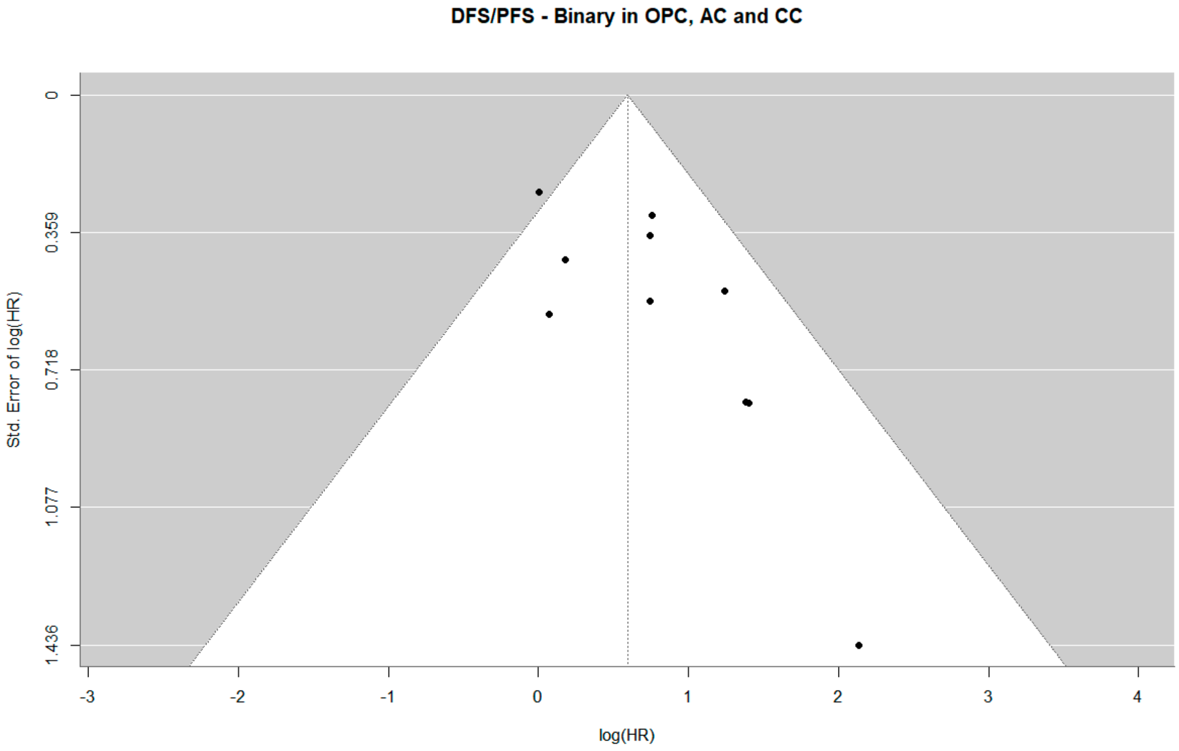

Supplementary Figure S8. Funnel plot for the association between pretreatment ctHPV (high vs low) and PFS/DFS.

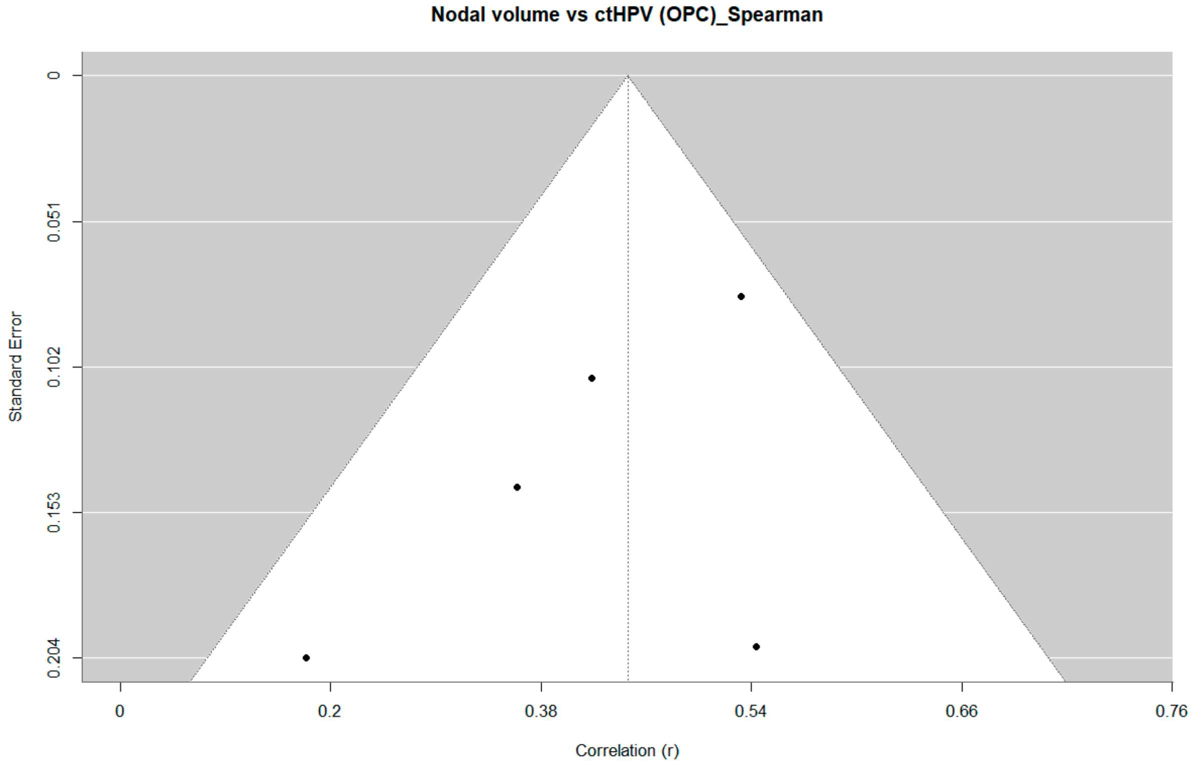

Supplementary Figure S9. Funnel plot for correlation between pretreatment ctHPV and GTV-N in OPC.

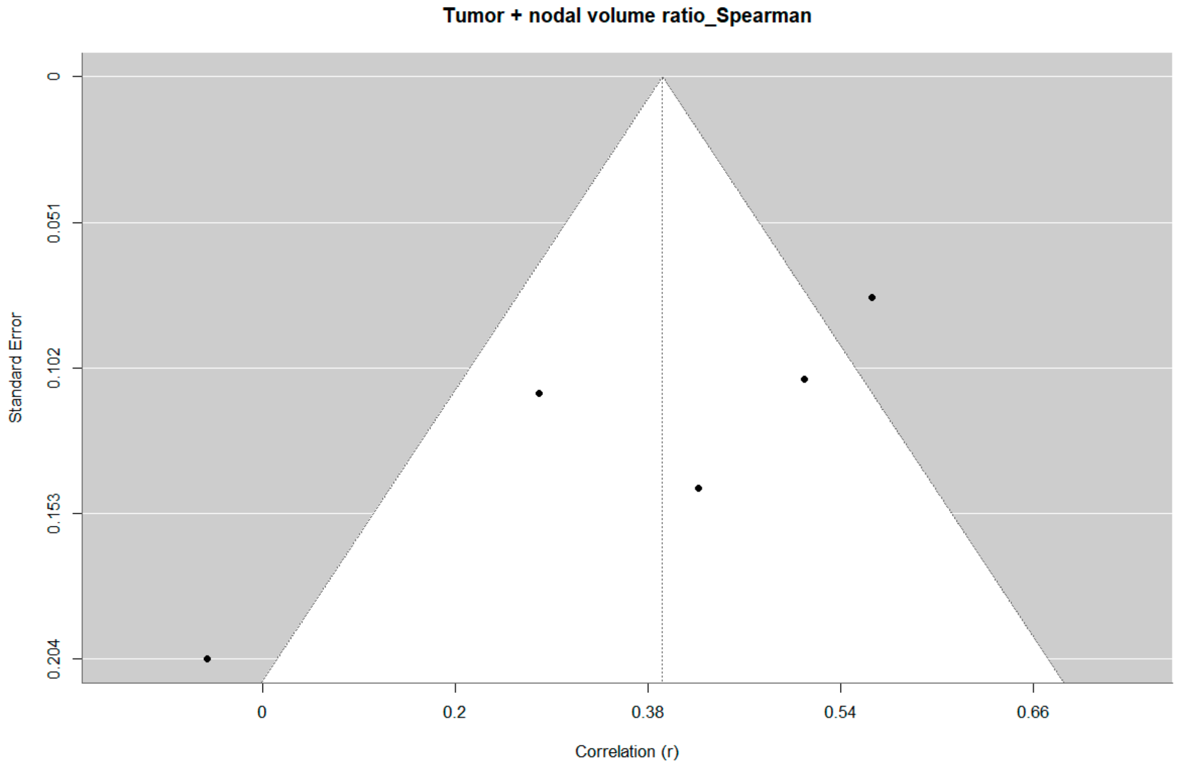

Supplementary Figure S10. Funnel plot for correlation between pretreatment ctHPV and GTV-T+N in OPC + AC.

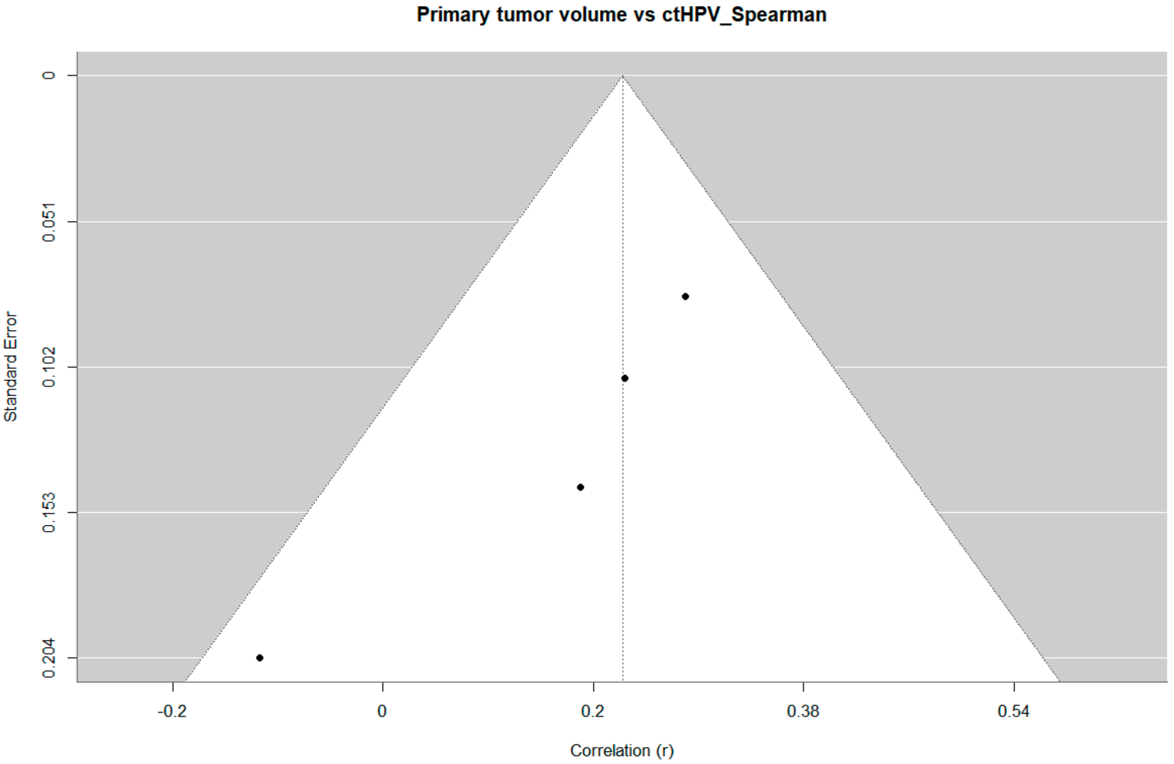

Supplementary Figure S11. Funnel plot for correlation between pretreatment ctHPV and GTV-T in OPC.

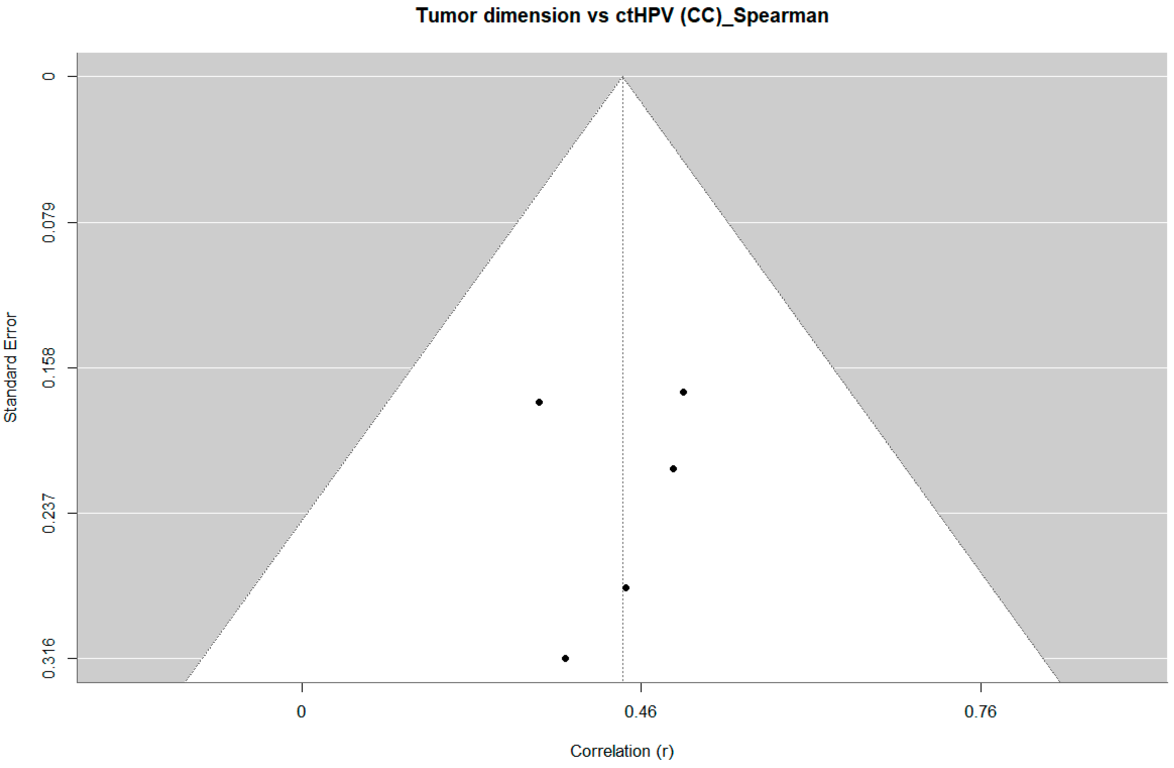

Supplementary Figure S12. Funnel plot for correlation between pretreatment ctHPV and tumor diameter in CC.

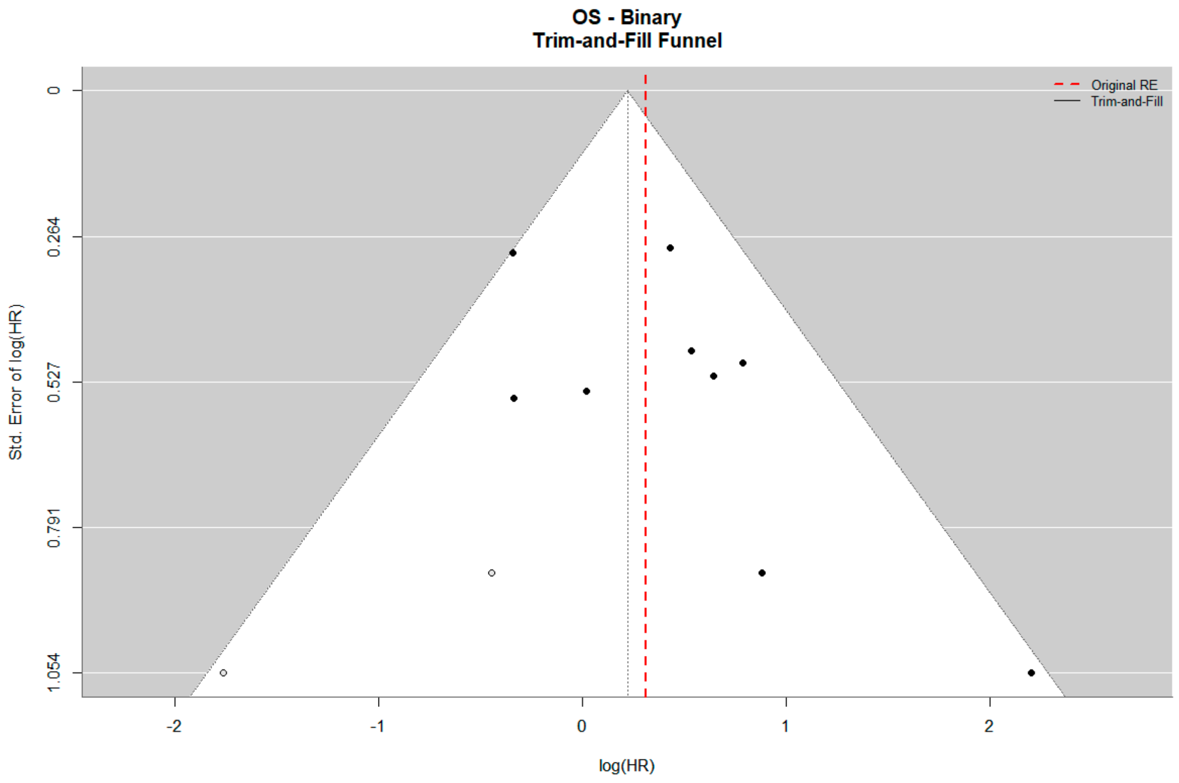

Supplementary Figure S13. Duval and Tweedie's Trim-and-Fill funnel plot for the association between pretreatment ctHPV (high vs low) and OS.

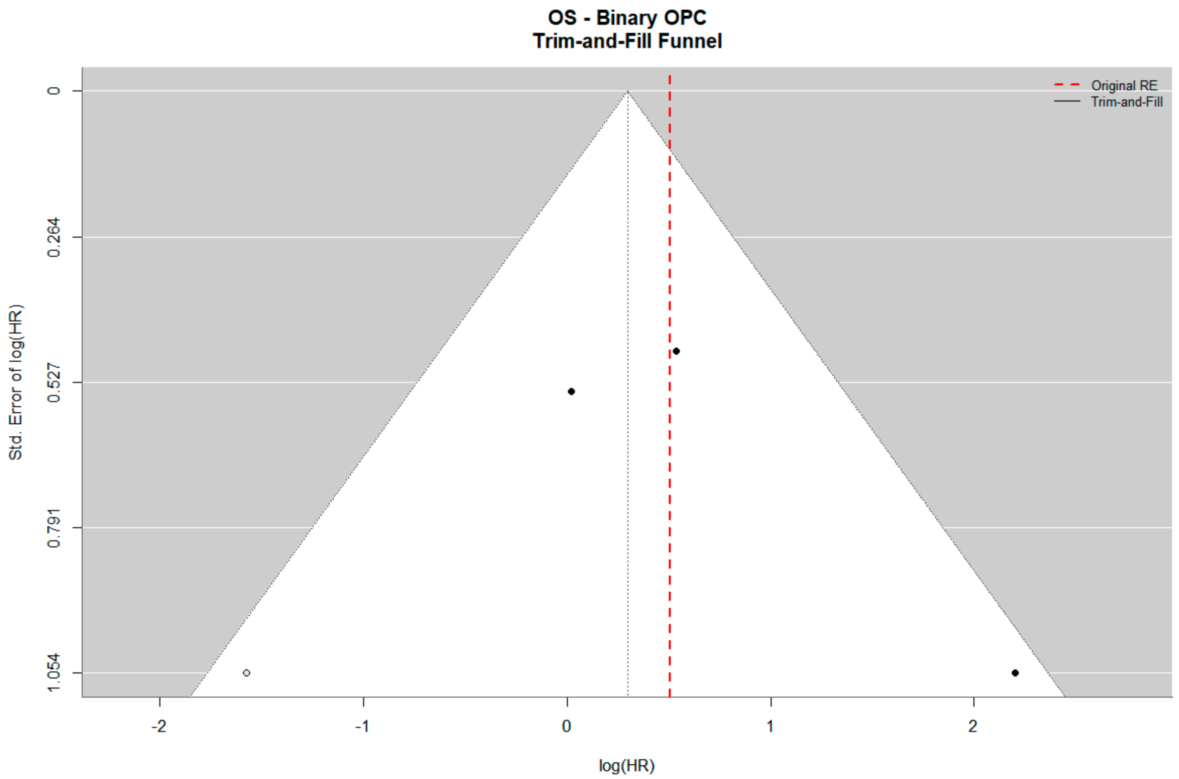

Supplementary Figure S14. Duval and Tweedie's Trim-and-Fill funnel plot for the association between pretreatment ctHPV (high vs low) and OS in the OPC subgroup.

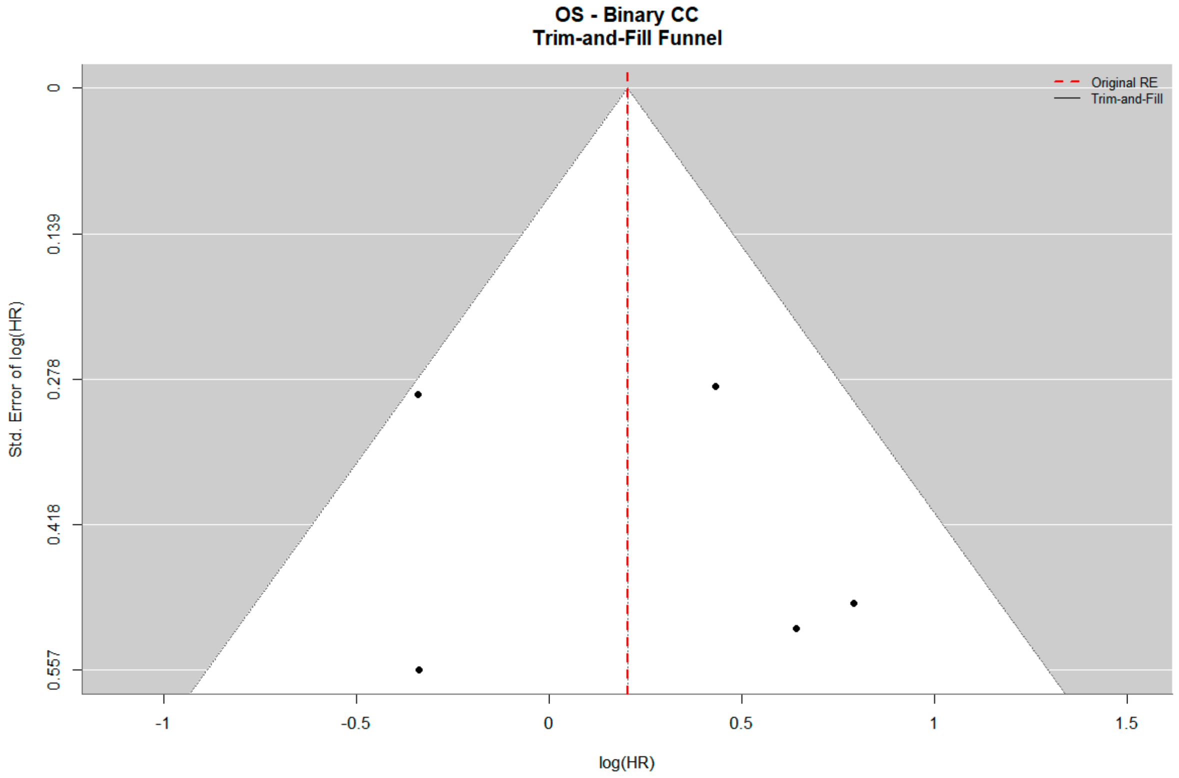

Supplementary Figure S15. Duval and Tweedie's Trim-and-Fill funnel plot for the association between pretreatment ctHPV (high vs low) and OS in the CC subgroup.

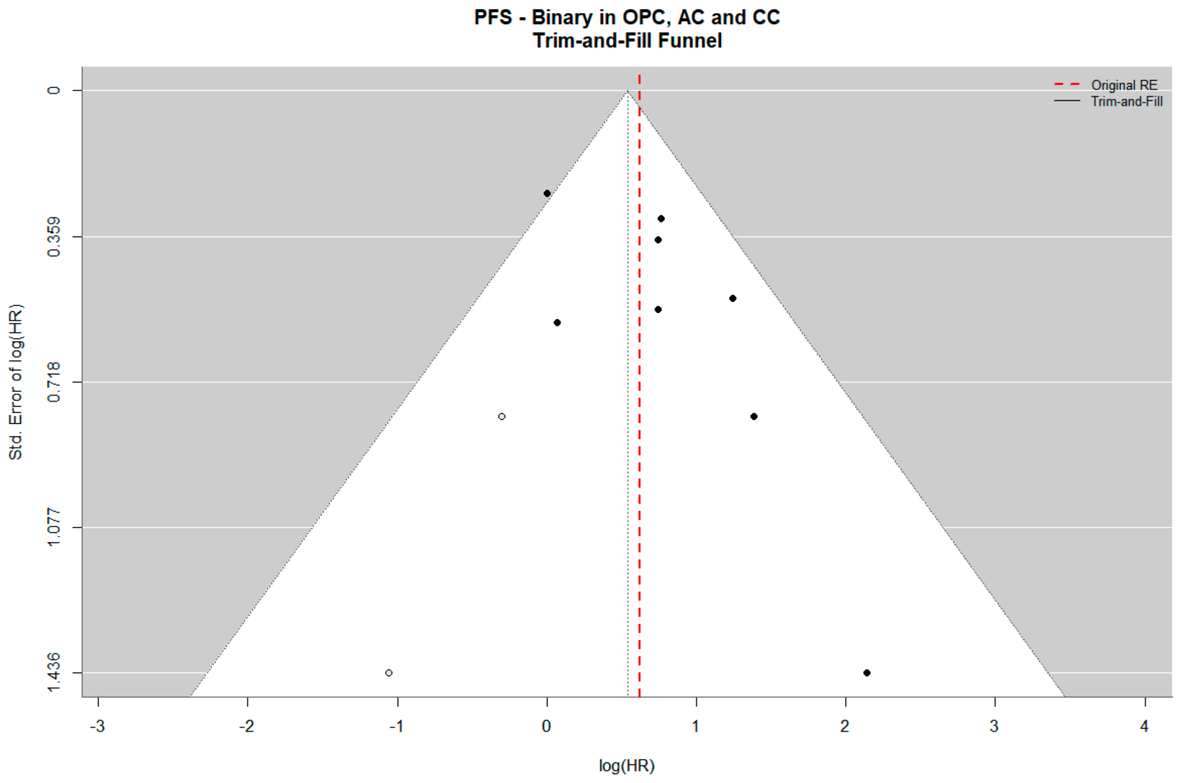

Supplementary Figure S16. Duval and Tweedie's Trim-and-Fill funnel plot for association between pretreatment ctHPV (high vs low) and PFS (OPC, AC, CC).

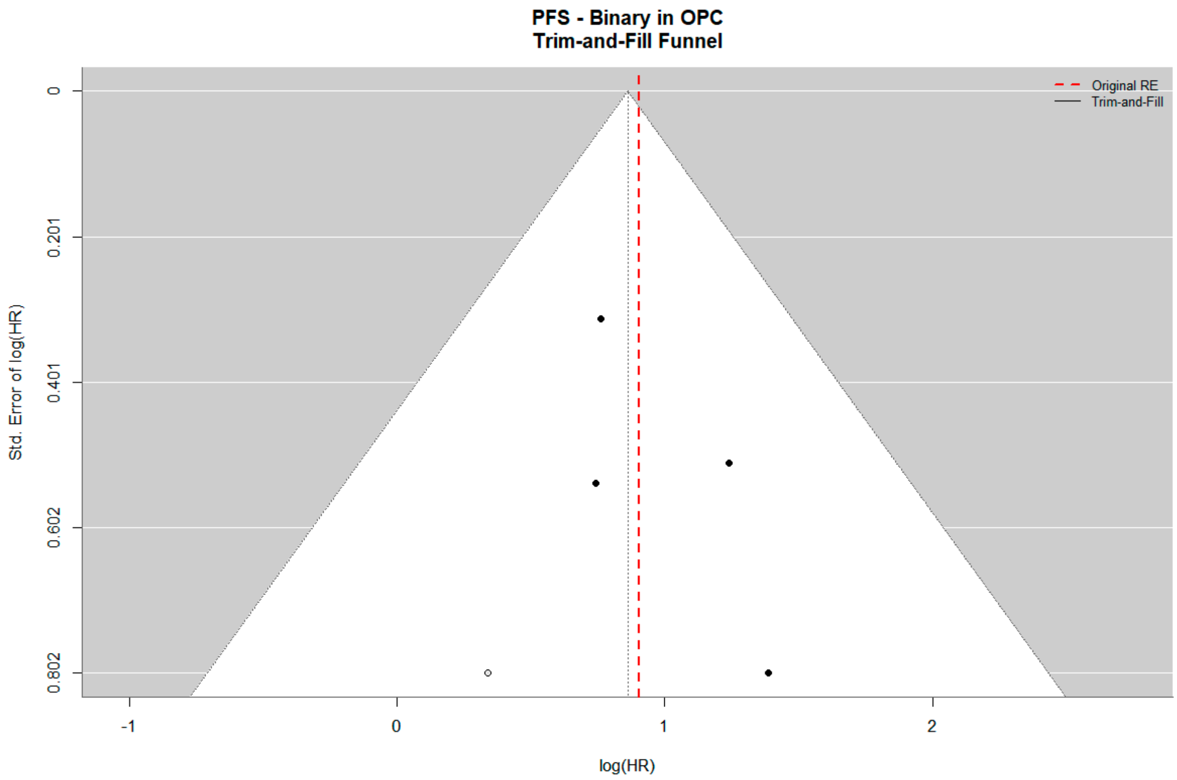

Supplementary Figure S17. Duval and Tweedie's Trim-and-Fill funnel plot for association between pretreatment ctHPV (high vs low) and PFS in OPC subgroup.

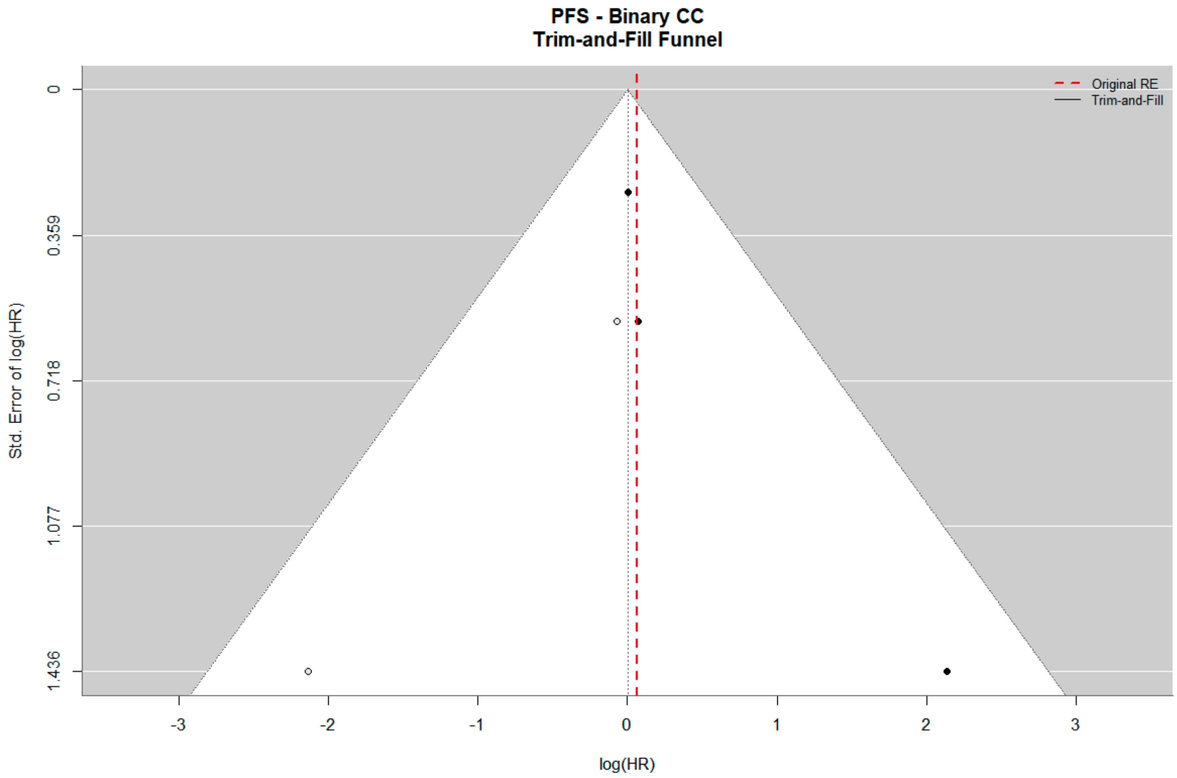

Supplementary Figure S18. Duval and Tweedie's Trim-and-Fill funnel plot for association between pretreatment ctHPV (high vs low) and PFS in CC subgroup.

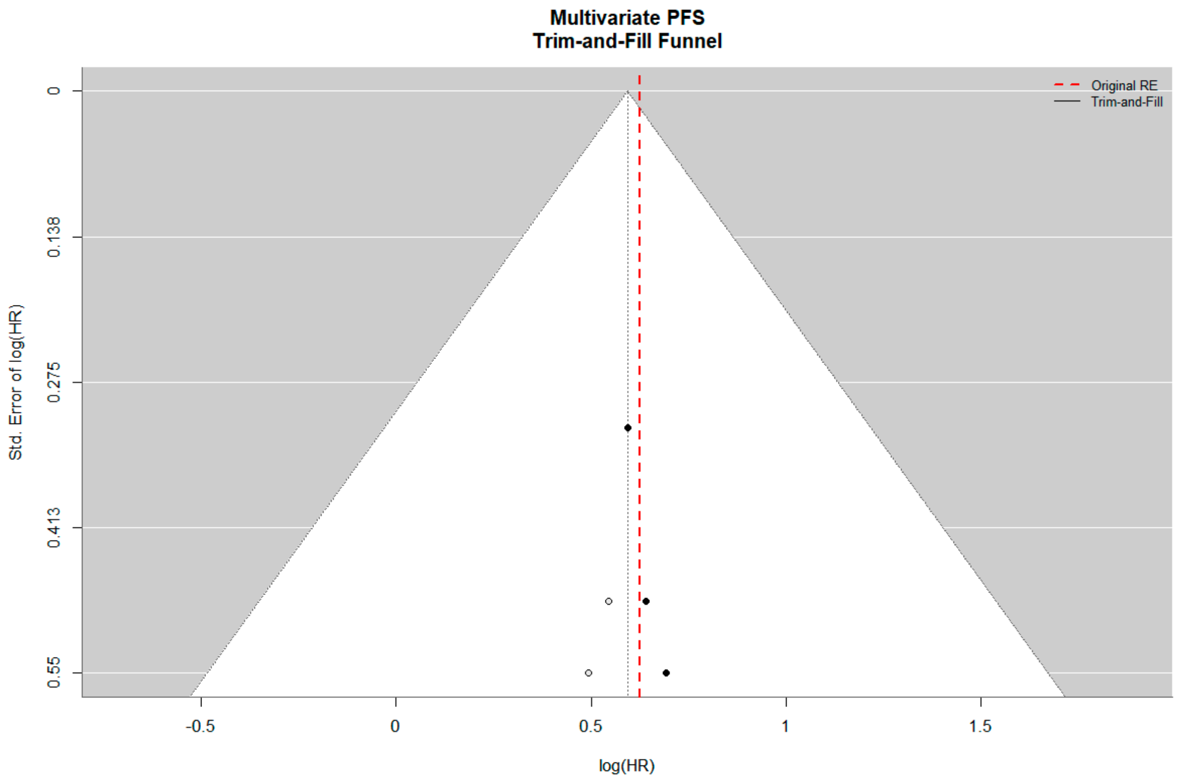

Supplementary Figure S19. Duval and Tweedie's Trim-and-Fill funnel plot for association between pretreatment ctHPV (high vs low) and PFS in OPC subgroup – multivariate analysis.

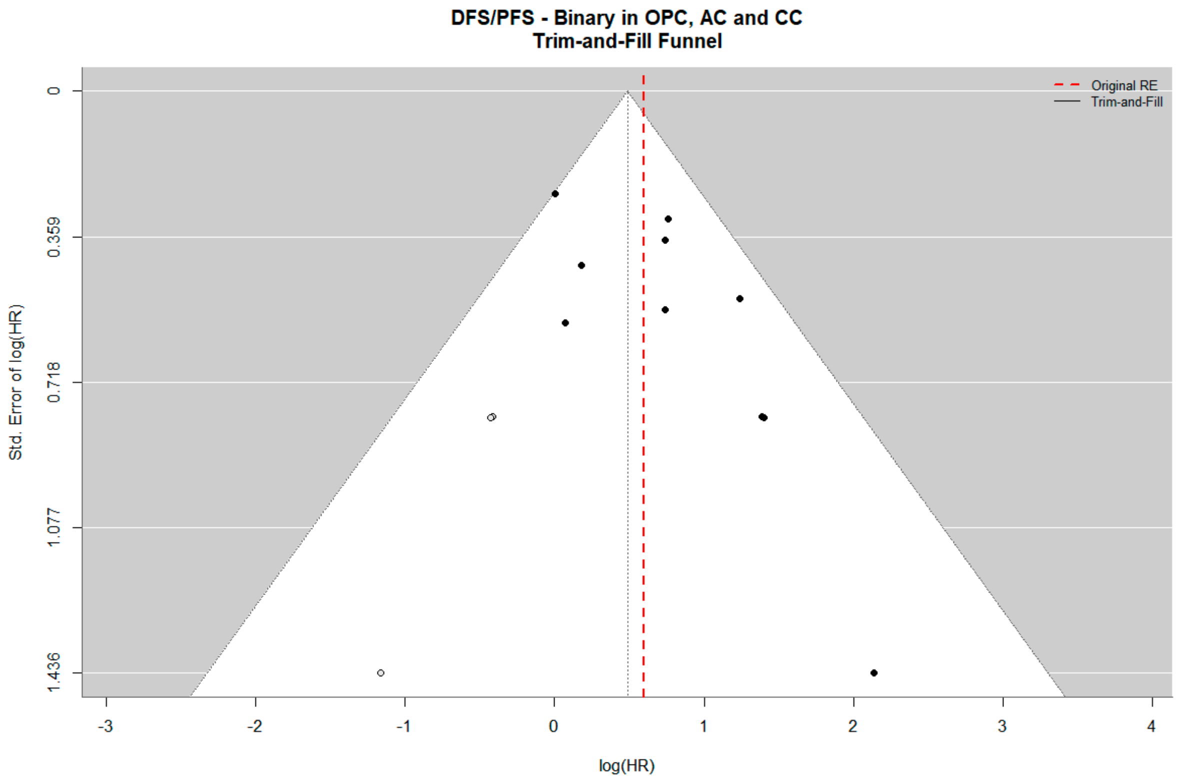

Supplementary Figure S20. Duval and Tweedie’s Trim-and-Fill funnel plot for association between pretreatment ctHPV (high vs low) and PFS/DFS (OPC, AC, CC).

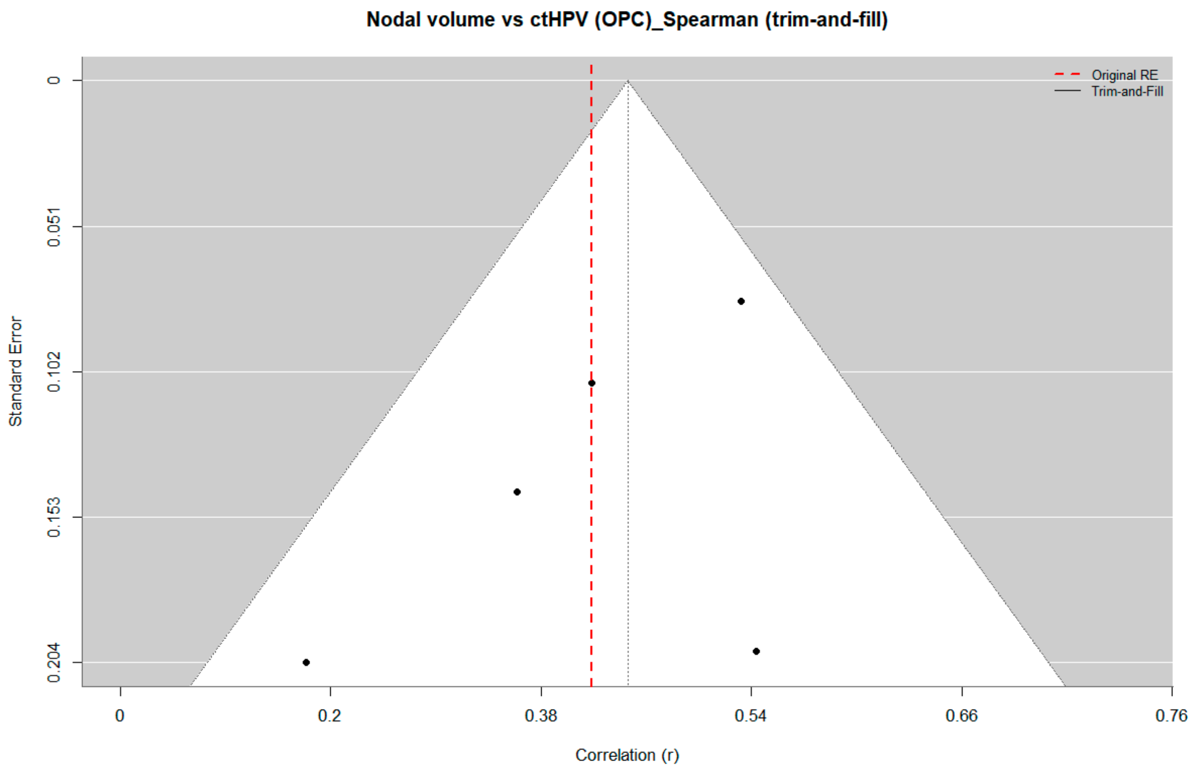

Supplementary Figure S21. Duval and Tweedie’s Trim-and-Fill funnel plot for correlation between pretreatment ctHPV and GTV-N in OPC.

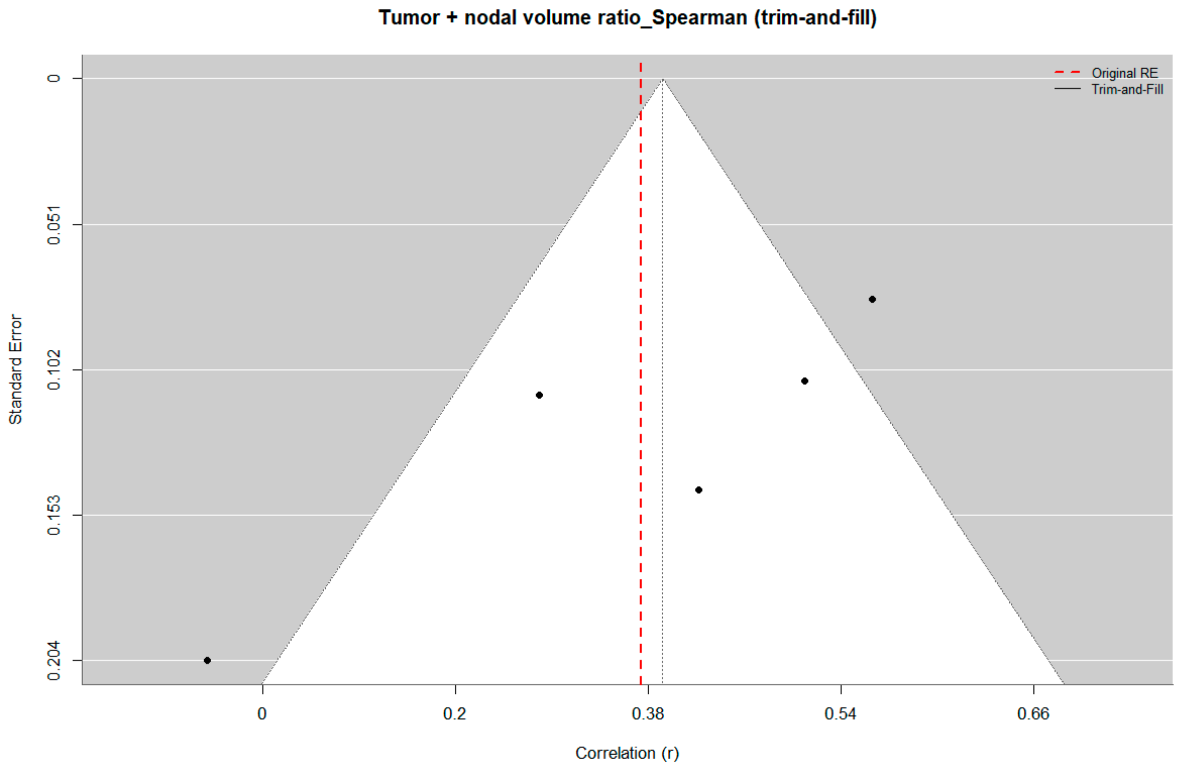

Supplementary Figure S22. Duval and Tweedie's Trim-and-Fill funnel plot for correlation between pretreatment ctHPV and GTV-T+N in OPC + AC.

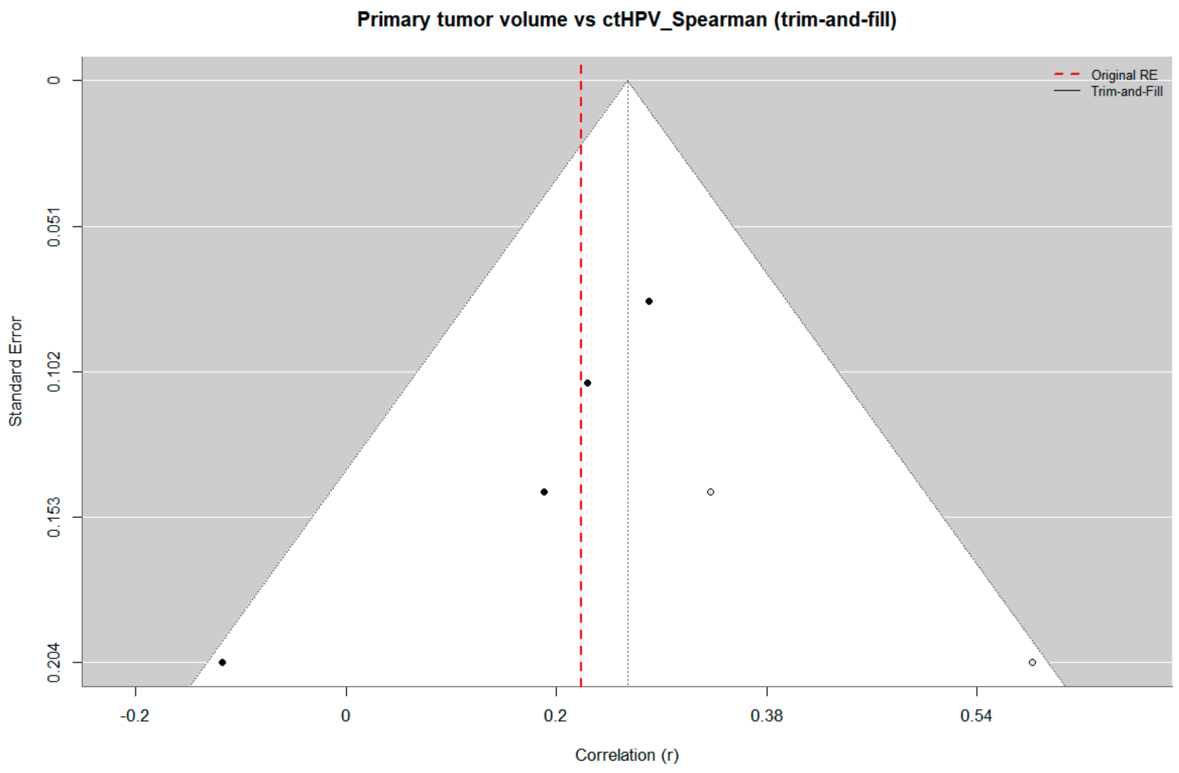

Supplementary Figure S23. Duval and Tweedie's Trim-and-Fill funnel plot for correlation between pretreatment ctHPV and GTV-T in OPC.

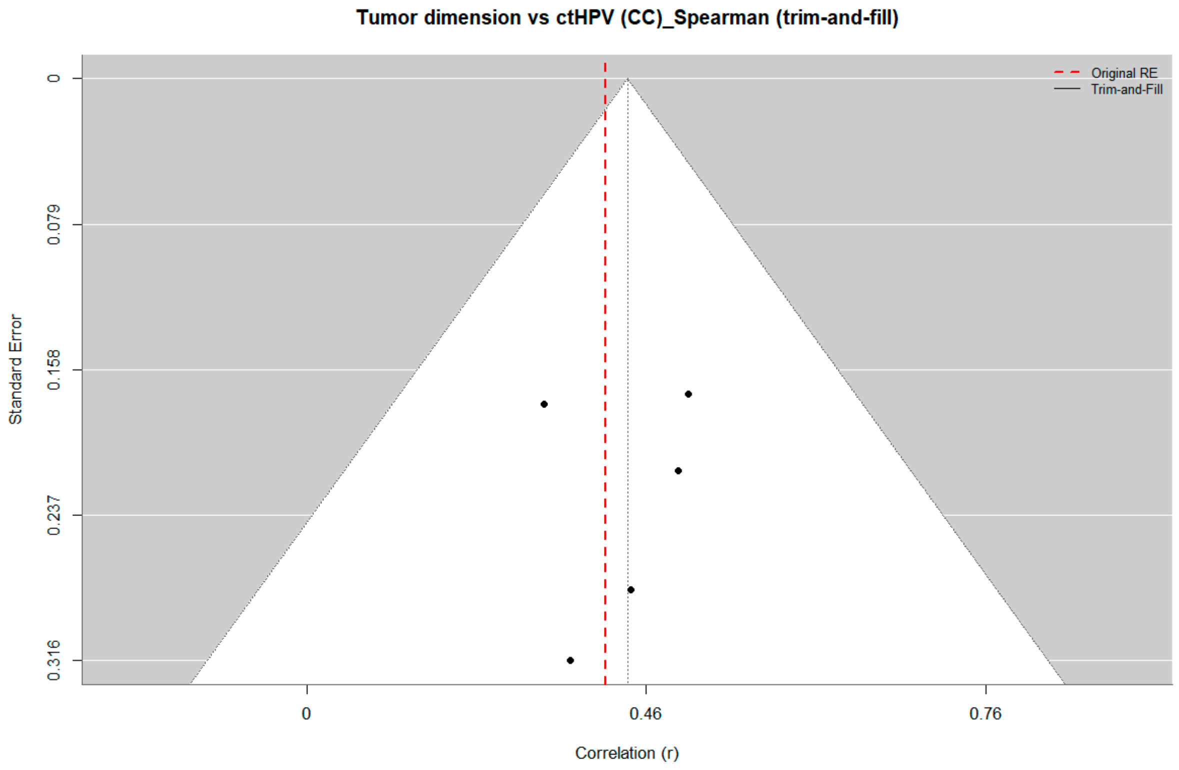

Supplementary Figure S24. Duval and Tweedie's Trim-and-Fill funnel plot for correlation between pretreatment ctHPV and tumor diameter in CC.
