# Supplementary material for: Prognostic Value of Pretreatment Circulating Tumor HPV DNA Load in HPV-Associated Cancers: A Systematic Review and Meta-Analysis
Source: Int J Mol Sci. 2026 May 11;27(10):4263. doi: 10.3390/ijms27104263 (PMC13207696; doi:10.3390/ijms27104263)
Supplement: Supplementary file 1 [file ijms-27-04263-s001.zip › Supplementary File S2.pdf]

PubMed

(Hpv[tiab] OR “human papilloma virus”[tiab:~2] OR “Human Papillomavirus Viruses”[Mesh]) AND (DNA OR cfDNA OR ctDNA OR cthpv OR cfhpv OR cthpv* OR cfhpv*) AND (tumor OR tumour OR neoplasm OR cancer OR carcinoma OR Neoplasms[Mesh]) AND (blood OR “Blood”[Mesh] OR serum OR “serum”[Mesh] OR plasma OR “plasma”[Mesh] OR circulat*) AND (human* OR “humans”[Mesh] OR men OR women OR person* OR “persons”[Mesh] OR people OR individual* OR patient* OR subjects) AND (anal OR anus OR “Anal Canal”[Mesh] OR “Anus Neoplasms”[Mesh] OR cervical OR cervix OR “Cervix Uteri”[Mesh] OR “Uterine Cervical Neoplasms”[Mesh] OR penis OR penile OR “Penis”[Mesh] OR “Penile Neoplasms”[Mesh] OR oropharynx OR oropharyngeal OR “Oropharyngeal Neoplasms”[Mesh] OR vulvar OR vulva OR Vulva[Mesh] OR “Vulvar Neoplasms”[Mesh] OR tongue OR Tongue[Mesh] OR glossal OR “Tongue Neoplasms”[Mesh] OR pharyngeal OR pharynx OR Pharynx[Mesh] OR “Pharyngeal Neoplasms”[Mesh] OR head OR Head[Mesh] OR neck OR Neck[Mesh] OR “Head and Neck Neoplasms”[Mesh])

Scopus

(TITLE-ABS-KEY(hpv OR hpv* OR *hpv OR *hpv* OR (human AND papilloma AND virus)) AND TITLE-ABS-KEY(tumor OR tumour OR neoplasm OR cancer OR carcinoma) AND TITLE-ABS-KEY(*dna OR dna OR cthpv OR cfhpv OR cthpv* OR cfhpv*) AND TITLE-ABS-KEY(blood OR plasma OR serum OR circulat*) AND TITLE-ABS-KEY(adult OR patient OR participant OR human OR men OR women OR subject) AND TITLE-ABS-KEY(anal OR anus OR cervical OR cervix OR penile OR penis OR oropharyngeal OR vulvar OR vulva OR tongue OR glossal OR pharynx OR head OR neck)) AND (EXCLUDE (DOCTYPE,"re"))

Embase

(hpv OR hpv* OR hpv OR hpv* OR (human AND papilloma AND virus)) AND (tumor OR tumour OR neoplasm OR cancer OR carcinoma OR tumors OR tumours OR neoplasms OR cancers OR carcinomas) AND (dna OR ctdna OR cfdna OR cthpv OR cfhpv OR cthpv* OR cfhpv*) AND ('blood'/exp OR blood OR 'serum'/exp OR serum OR 'plasma'/exp OR plasma OR circulat*) AND (human* OR 'men'/exp OR men OR 'women'/exp OR women OR person* OR people OR individual* OR patient* OR subjects) AND (anal OR anus OR ‘anus’/exp OR cervical OR cervix OR ‘cervix’/exp OR penile OR penis OR ‘penis’/exp OR oropharyngeal OR ‘oropharynx’/exp OR vulvar OR vulva OR ‘vulva’/exp OR tongue OR ‘tongue’/exp OR glossal OR pharynx OR ‘pharynx’/exp OR head OR neck)

Cochrane

(hpv OR hpv* OR *hpv OR *hpv* OR (human AND papilloma AND virus)) AND (tumor OR tumour OR neoplasm OR cancer OR carcinoma) AND (*dna OR dna OR cthpv OR cfhpv OR cthpv* OR cfhpv*) AND (blood OR plasma OR serum OR circulat*) AND (adult OR patient OR participant OR human OR men OR women OR subject) AND (anal OR anus

OR cervical OR cervix OR penile OR penis OR oropharyngeal OR vulvar OR vulva OR tongue OR glossal OR pharynx OR head OR neck)

Web of Science

(TS=(hpv OR hpv* OR (human AND papilloma AND virus)) OR TI=(hpv OR hpv* OR (human AND papilloma AND virus)) OR AB=(hpv OR hpv* OR (human AND papilloma AND virus)) OR AK=(hpv OR hpv* (human AND papilloma AND virus)) OR KP=(hpv OR hpv* OR (human AND papilloma AND virus)) OR SU=(hpv OR hpv* OR (human AND papilloma AND virus)) OR WC=(hpv OR hpv* OR (human AND papilloma AND virus))) AND (TS=(tumor OR tumour OR neoplasm OR cancer OR carcinoma) OR TI=(tumor OR tumour OR neoplasm OR cancer OR carcinoma) OR AB=(tumor OR tumour OR neoplasm OR cancer OR carcinoma) OR AK=(tumor OR tumour OR neoplasm OR cancer OR carcinoma) OR KP=(tumor OR tumour OR neoplasm OR cancer OR carcinoma) OR SU=(tumor OR tumour OR neoplasm OR cancer OR carcinoma) OR WC=(tumor OR tumour OR neoplasm OR cancer OR carcinoma)) AND (TS=(ctdna OR cfdna OR dna OR cthpv OR cfhpv OR cthpv* OR cfhpv*) OR TI=(ctdna OR cfdna OR dna OR cthpv OR cfhpv OR cthpv* OR cfhpv*) OR AB=(ctdna OR cfdna OR dna OR cthpv OR cfhpv OR cthpv* OR cfhpv*) OR AK=(ctdna OR cfdna OR dna OR cthpv OR cfhpv OR cthpv* OR cfhpv*) OR KP=(ctdna OR cfdna OR dna OR cthpv OR cfhpv OR cthpv* OR cfhpv*) OR SU=(ctdna OR cfdna OR dna OR cthpv OR cfhpv OR cthpv* OR cfhpv*) OR WC=(ctdna OR cfdna OR dna OR cthpv OR cfhpv OR cthpv* OR cfhpv*)) AND (TS=(anal OR anus OR cervical OR cervix OR penile OR penis OR oropharyngeal OR vulvar OR vulva OR tongue OR glossal OR pharynx OR head OR neck) OR TI=(anal OR anus OR cervical OR cervix OR penile OR penis OR oropharyngeal OR vulvar OR vulva OR tongue OR glossal OR pharynx OR head OR neck) OR AB=(anal OR anus OR cervical OR cervix OR penile OR penis OR oropharyngeal OR vulvar OR vulva OR tongue OR glossal OR pharynx OR head OR neck) OR AK=(anal OR anus OR cervical OR cervix OR penile OR penis OR oropharyngeal OR vulvar OR vulva OR tongue OR glossal OR pharynx OR head OR neck) OR KP=(anal OR anus OR cervical OR cervix OR penile OR penis OR oropharyngeal OR vulvar OR vulva OR tongue OR glossal OR pharynx OR head OR neck) OR SU=(anal OR anus OR cervical OR cervix OR penile OR penis OR oropharyngeal OR vulvar OR vulva OR tongue OR glossal OR pharynx OR head OR neck) OR WC=(anal OR anus OR cervical OR cervix OR penile OR penis OR oropharyngeal OR vulvar OR vulva OR tongue OR glossal OR pharynx OR head OR neck)) AND (TS=(adult OR patient OR participant OR human OR men OR women OR subject) OR TI=(adult OR patient OR participant OR human OR men OR women OR subject) OR AB=(adult OR patient OR participant OR human OR men OR women OR subject) OR AK=(adult OR patient OR participant OR human OR men OR women OR subject) OR KP=(adult OR patient OR participant OR human OR men OR women OR subject) OR SU=(adult OR patient OR participant OR human OR men OR women OR subject) OR WC=(adult OR patient OR participant OR human OR men OR women OR subject)) AND (TS=(blood OR serum OR

plasma OR circulat*) OR TI=(blood OR serum OR plasma OR circulat*) OR AB=(blood OR serum OR plasma OR circulat*) OR AK=(blood OR serum OR plasma OR circulat*) OR KP=(blood OR serum OR plasma OR circulat*) OR SU=(blood OR serum OR plasma OR circulat*) OR WC=(blood OR serum OR plasma OR circulat*))

AND (TS=(blood OR serum OR plasma) OR TI=(blood OR serum OR plasma) OR AB=(blood OR serum OR plasma) OR AK=(blood OR serum OR plasma) OR KP=(blood OR serum OR plasma) OR SU=(blood OR serum OR plasma) OR WC=(blood OR serum OR plasma))

AND (TS=() OR TI=() OR AB=() OR AK=() OR KP=() OR SU=() OR WC=())

(TS=(hpv OR hpv* OR (human AND papilloma AND virus)) OR TI=(hpv OR hpv* OR (human AND papilloma AND virus)) OR AB=(hpv OR hpv* OR (human AND papilloma AND virus)) OR AK=(hpv OR hpv* (human AND papilloma AND virus)) OR KP=(hpv OR hpv* OR (human AND papilloma AND virus)) OR SU=(hpv OR hpv* OR (human AND papilloma AND virus)) OR WC=(hpv OR hpv* OR (human AND papilloma AND virus)))

AND (TS=(tumor OR tumour OR neoplasm OR cancer OR carcinoma) OR TI=(tumor OR tumour OR neoplasm OR cancer OR carcinoma) OR AB=(tumor OR tumour OR neoplasm OR cancer OR carcinoma) OR AK=(tumor OR tumour OR neoplasm OR cancer OR carcinoma) OR KP=(tumor OR tumour OR neoplasm OR cancer OR carcinoma) OR SU=(tumor OR tumour OR neoplasm OR cancer OR carcinoma) OR WC=(tumor OR tumour OR neoplasm OR cancer OR carcinoma))

(TS=(ctdna OR cfdna OR dna OR cthpv OR cfhpv OR cthpv* OR cfhpv*) OR TI=(ctdna OR cfdna OR dna OR cthpv OR cfhpv OR cthpv* OR cfhpv*) OR AB=(ctdna OR cfdna OR dna OR cthpv OR cfhpv OR cthpv* OR cfhpv*) OR AK=(ctdna OR cfdna OR dna OR cthpv OR cfhpv OR cthpv* OR cfhpv*) OR KP=(ctdna OR cfdna OR dna OR cthpv OR cfhpv OR cthpv* OR cfhpv*) OR SU=(ctdna OR cfdna OR dna OR cthpv OR cfhpv OR cthpv* OR cfhpv*) OR WC=(ctdna OR cfdna OR dna OR cthpv OR cfhpv OR cthpv* OR cfhpv*))

AND (TS=(anal OR anus OR cervical OR cervix OR penile OR penis OR oropharyngeal OR vulvar OR vulva OR tongue OR glossal OR pharynx OR head OR neck) OR TI=(anal OR anus OR cervical OR cervix OR penile OR penis OR oropharyngeal OR vulvar OR vulva OR tongue OR glossal OR pharynx OR head OR neck) OR AB=(anal OR anus OR cervical OR cervix OR penile OR penis OR oropharyngeal OR vulvar OR vulva OR tongue OR glossal OR pharynx OR head OR neck) OR AK=(anal OR anus OR cervical OR cervix OR penile OR penis OR oropharyngeal OR vulvar OR vulva OR tongue OR glossal OR pharynx OR head OR neck) OR KP=(anal OR anus OR cervical OR cervix OR penile OR penis OR oropharyngeal OR vulvar OR vulva OR tongue OR glossal OR pharynx OR head OR neck) OR SU=(anal OR anus OR cervical OR cervix OR penile OR penis OR oropharyngeal OR vulvar OR vulva OR tongue OR glossal OR pharynx OR head OR neck) OR WC=(anal OR anus OR cervical OR cervix OR penile OR penis OR oropharyngeal OR vulvar OR vulva OR tongue OR glossal OR pharynx OR head OR neck))

AND (TS=(adult OR patient OR participant OR human OR men OR women OR subject) OR TI=(adult OR patient OR participant OR human OR men OR women OR subject) OR AB=(adult OR patient OR participant OR human OR men OR women OR subject) OR AK=(adult OR patient OR participant OR human OR men OR women OR subject) OR KP=(adult OR patient OR participant OR human OR men OR women OR subject) OR SU=(adult OR patient OR participant OR human OR men OR women OR subject) OR WC=(adult OR patient OR participant OR human OR men OR women OR subject))

AND (TS=(blood OR serum OR plasma OR circulat*) OR TI=(blood OR serum OR plasma OR circulat*) OR AB=(blood OR serum OR plasma OR circulat*) OR AK=(blood OR serum OR plasma OR circulat*) OR KP=(blood OR serum OR plasma OR circulat*) OR SU=(blood OR serum OR plasma OR circulat*) OR WC=(blood OR serum OR plasma OR circulat*))
