# Supplementary material for: Prognostic Value of Pretreatment Circulating Tumor HPV DNA Load in HPV-Associated Cancers: A Systematic Review and Meta-Analysis
Source: Int J Mol Sci. 2026 May 11;27(10):4263. doi: 10.3390/ijms27104263 (PMC13207696; doi:10.3390/ijms27104263)
Supplement: Supplementary file 1 [file ijms-27-04263-s001.zip › Supplementary Table S1.pdf]

Study (Author, Year)	Country	Cancer Type[a]	Number of patients[b]	HPV Type	Method	Sample Timing	ctHPV scale	Endpoints	Follow-up[c]	Disease Stage	Association of ctHPV level with survival outcomes[d]	Association of ctHPV level with quantitative tumor burden metrics[d,e]
Studies included in quantitative synthesis												
**Mazurek et al., 2024**	Poland	OPC	91	HPV16	qPCR	Pre-treatment	Continuous; Binary with ROC-derived cut-off (3.556 log_{10} copies/mL),	MFS, LRFS, OS, PFS*	Median 45 months	T1-4, N0-3, M0 (AJCC 8)	Univariate:<br>OS continuous (HR 1.08, p = 0.779)*<br>OS high/low (HR 1.02, p = 0.975)*;<br>PFS (2.10, p = 0.170)*;<br>Multivariate:<br>MFS continuous (HR 2.22, p = 0.015);<br>MFS high/low (HR 5.5, p = 0.033);<br>OS continuous (HR 1.12, p = 0.7);<br>LRFS continuous (HR 1.12, p = 0.705);<br>PFS high/low (2.00, p = 0.208)*;	NR
**Adrian et al., 2023**	Sweden	OPC	108	HPV16	qPCR	Pre-treatment; end of treatment	Continuous; binary with cut-off based on median (67,5 copies/mL)	PFS, OS	Median 33.6 months	Stage III-IV (UICC 7)	Univariate:<br>PFS continuous (HR 1.26, p = 0.01);<br>PFS high/low (HR 3.46, p = 0.015)**;<br>OS continuous (HR 1.38, p = 0.013);<br>OS high/low (HR 9.04, p = 0.037)**;<br>Multivariate:<br>Not performed	GTV-T+N (Pearson's r = 0.39, p <0.001)
**Wotman et al., 2025**	USA	OPC, HNCUP	221	HPV16, 18, 31, 33, 35, 39, 45, 51, 52, 56, 58, 59, 68	ddPCR	Pre-treatment	Binary with cut-off based on median (231 copies/mL)	PFS, OS	Median 26.4 months	Stage I-IVB (AJCC 7)	Univariate:<br>PFS high/low (HR 2.14, p = 0.0156);<br>OS high/low (HR 1.71, p = 0.2728)**<br>Multivariate:<br>PFS high/low (HR 1.81, p = 0.0635);	NR
**Ecchevaria et al., 2025**	USA	OPC	48	HPV16, 18, 31, 33, 35	NavDx (ddPCR)	Pre-treatment, mid-treatment, end of treatment, follow-up	Binary with cut-off based on median (132 fragments/mL)	PFS	Median 41 months	T0-2, N0-1, M0	Univariate:<br>PFS high/low (HR 4.00, p = 0.08)**<br>Multivariate:<br>Not performed	NR
**Bernard-Tessier et al., 2019**	France	AC	57	HPV16	ddPCR	Pre-CHT; end-CHT	Binary with ROC-derived cut-off (2940 copies/mL)	PFS	Not specified	Metastatic or locoregion al recurrence	Univariate:<br>PFS (HR 2.1, p = 0.04);<br>Multivariate:<br>PFS (HR 1.9, p = 0.18);	sum of diameters of RECIST target lesions (Spearman's ρ = 0.32; p = 0.025)

**Lefèvre et al., 2021**	Denmark	AC	45	HPV16, 18, 31, 33, 51, 58	ddPCR	Pre-treatment, mid-treatment, end of treatment, follow-up	Binary with cut-off based on median (1.34% of total cfDNA)	DFS, OS	Median 29 months	T1–4, N+/–, M0–1	Univariate:<br>OS high/low (HR 2.42, p = 0.31);<br>DFS high/low (HR 4.07, p = 0.08);<br>Multivariate:<br>Not performed<br>n = 45	NR
**Cabel et al., 2021**	France	CC	55	HPV16,18,31,33,35,45,52,58,73	ddPCR	Pre-treatment, end of treatment, follow-up	Binary with cut-off based on median (33 copies/mL)	DFS, OS	Median 49,9 months (retrospective cohort); 37 months (prospective cohort)	FIGO I–IVA	Univariate:<br>OS high/low (HR = 1.9, p = 0.24);<br>DFS high/low (HR = 1.2, p = 0.67);<br>Multivariate:<br>Not performed	NR
**Kim et al., 2022**	South Korea	CC	14	HPV16, 18	NGS	Pre-treatment, during RT, 3 months after RT	Binary with cut-off based on mean (0.13607% of total cfDNA)	PFS	Median 25.4 months	FIGO IB–IIIC	Univariate:<br>PFS high/low (HR = 8.49, p=0.16)**.<br>Multivariate:<br>Not performed	NR
**Cheung et al., 2019**	China (Hong Kong)	CC	138	HPV16, 18	ddPCR	Pre-treatment	Cut-off 20 copies/20 ul (1000 copies/mL)	OS, PFS	Not specified	FIGO I-IV	Univariate:<br>OS high/low (RR = 1.70, p = 0.007) (HR = 1.54, p = 0.133)**<br>PFS high/low (RR = 1.69, p = 0.030);<br>Multivariate:<br>no significant associations	NR
**Mayadev et al., 2025**	India	CC	185	HPV16, 18	ddPCR	Pretreatment, follow-up	Binary with cut-off based on median 54.6 copies/mL	OS, PFS	Median 37.8 months	FIGO IB2-IIB node-positive or IIIA-IVA any node-status	Univariate:<br>PFS high/low (HR 1.00, p=0.99)****<br>OS high/low (HR 0.71, p=0.25)****<br>Multivariate:<br>Not Performed	NR

**Collier et al., 2025**	Canada	CC	21	HPV16, 18, 31, 33, 59	NGS, ddPCR	Pretreatment, mid-treatment, end of treatment, follow-up	Binary with cut-off based on median 299 copies/mL	PFS, OS	Not specified	Metastatic or recurrent	Univariate:<br>OS high/low (HR 2.20, p = 0.10)**<br>PFS high/low (HR 1.08, p = 0.90)**<br>Multivariate:<br>Not performed	NR
**Yin et al., 2025**	China	CC	28	HPV16, 18, 31, 33, 35, 39, 45, 51, 52, 56, 58, 59, 68.	ddPCR	Pretreatment, follow-up	Binary with cut-off based on median 3.9×10^4 copies/mL	OS	Median 42.3 months	Metastatic or recurrent	Univariate:<br>OS high/low (HR 0.714, p = 0.55)**<br>Multivariate:<br>Not performed<br>(Kendall's τ correlation R^2=–0.037, p=0.782,).	NR
**Cao et al., 2021**	USA	OPC	28	HPV16, 18	ddPCR	Pre-treatment, during CRT (2, 4 and 7 week), post-treatment	NA	FFP	Mean 28 months	Stage III (AJCC 8)	Univariate:<br>FFP (HR=1.06, p<0.03) - ctHPV analyzed per 1000 copies/ml<br>Multivariate:<br>No significant associations (p=0.056)	GTV-N (Spearman's ρ = 0.54, p = 0.016)<br>MTV50-N (Spearman's ρ = 0.58, p = 0.016)<br>GTV-T, MTV50-T - no significant correlation (p > 0.300)
**Kentnowski et al., 2023**	Poland	OPC	51	HPV16	qPCR	Pre-treatment	NA	-	NA	T1-4, N1-3, M0 (AJCC 8)	NR	GTV-T (Spearman's ρ = 0.186, p = 0.187);<br>GTV-N (Spearman's ρ = 0.360, p = 0.009),<br>GTV-T+N (Spearman's ρ = 0.424, p = 0.002);
**Lee et al., 2017**	United Kingdom	HNSCC	27	HPV16	NGS	Pre-treatment, 6 and 12-week after treatment	NA	-	3 months	Stage III–IV (AJCC 2007)	NR	GTV-T (Spearman's ρ = -0.117, p = 0.471)<br>GTV-N (Spearman's ρ = 0.175, p = 0.287)<br>GTV-T+N (Spearman's ρ = 0.057, p = 0.722)
**Campo et al., 2024**	Italy	OPC	92	HPV16, 33, 35	ddPCR	Pre-treatment, repeatedly during follow-up	NA	-	Not specified	T0-4, N0-3, M0 (AJCC 8)	NR	GTV-N (Spearman's ρ = 0.42, p = 0.004)<br>GTV-T+N (Spearman's ρ = 0.51, p < 0.001)<br>GTV-T (Spearman's ρ = 0.226, p > 0.05)

**Damerla et al., 2020**	USA	OPC, AC	84	HPV16, 33	ddPCR, qPCR	Pre-treatment, repeatedly during treatment	NA	-	Not specified	Stage I-IVB (AJCC 7)	NR	GTV-T+N (Spearman's ρ = 0.28, p = 0.009)
**Campitelli et al., 2012**	France	CC	16	HPV16, 18	qPCR	Pre-treatment, follow-up in 2 patients	NA	-	Not specified	FIGO IB–IVA	NR	Tumor diameter (cm, Spearman's ρ = 0.4442, p = 0.08476) *
**Ho et al., 2005**	Taiwan	CC	13	HPV16, 18, 52	qPCR	Pre-treatment, follow-up (3-month intervals)	NA	-	>12 months	FIGO IB–IVB	NR	Tumor diameter (cm, Spearman's ρ = 0.37125, p = 0.06187)*
**Jeannot et al., 2016**	France	CC	37	HPV16, 18	ddPCR, qPCR	Pre-treatment	NA	-	NA	FIGO I–IV	NR	Tumor diameter (mm, Spearman's ρ = 0.51, p = 0.001)
**Bakhtiar et al., 2025**	USA	OPC	170	HPV16, 18, 31, 33, 35	NavDx (ddPCR)	Pre-treatment	NA	-	NA	Stages I-III	NR	GTV-T (Spearman's ρ = 0.28, p < 0.001)<br>GTV-N (Spearman's ρ = 0.530, p < 0.001)<br>GTV-T+N (Spearman's ρ = 0.560, p < 0.001)
**Gupta et al., 2025**	India	CC	60	HPV16, 18	ddPCR	Pre-treatment, 3 months after treatment	NA	-	3 months	FIGO I-IV	NR	Tumor diameter (cm, Spearman's ρ = 0.336, p = 0.048)
**Chen et al., 2025**	China	CC	25	HPV16, 18, 33, 52, 58	ddPCR	Pre-treatment	NA	-	NA	FIGO IA1–IVA	NR	Tumor diameter (mm, Spearman's ρ = 0.4979, p = 0.01)*
**Studies included in qualitative synthesis**												

**Sanz-Garcia et al., 2024**	Canada	HNSCC	29	HPV 16, 35	dPCR, NGS	Pre-treatment; follow-up 4–6 week, and 8–12 week after treatment	Continuous	RFS	Median 25 months	Stage III–IVC	RFS continuous (HR: 1.00, p = 0.31)	NR
**Califano et al., 2023**	United Kingdom	OPC	233	HPV16	qPCR	Pre-treatment, post-treatment	Continuous	RPFS	Median 25 months	Stage III-IVA (AJCC 7)	Univariate:<br>RPFS (HR, 4.78; 95% CI, 3.49-6.56)<br>Multivariate:<br>Not performed	NR
**Hanna et al., 2018**	USA	OPC	22	HPV16, 18, 31, 33, 45	ddPCR	Follow-up	Continuous	OS	Median 26.5 months	Metastatic or recurrent	OS (Kendall's τ R = −0.48, P=0.05)	TTB (Kendals τ = 0.91, p < 0.001)
**Sivars et al., 2024**	Sweden	CC	53	HPV16, 18, 31, 33, 35, 39, 45, 51, 52, 56, 58, 59, 66	ddPCR	Pre-treatment, during treatment, early (1-2 months) and late (>4 moths) follow-up	Tertiles based on ctHPV level	PFS	Median 37 months	FIGO I-IV	No significant association with PFS (p = 0.349) – cut-offs based on tertiles of ctHPV level	NR
**Cooke et al., 2025**	USA	OPC	203	HPV16, 18, 31, 33, 35	NavDx (ddPCR)	Pretreatment	Tertiles based on ctHPV level	RFS, DSS	Median 13 months	T0-4, N0-3, M0-1 (AJCC 8)	No significant association with RFS (p = 0.88) or DSS (p = 0.71) – cut-offs based on tertiles of ctHPV level	GTV-T+N (aOR 1.04; 95% CI 1.02-1.07)<br>SUVmax (aOR, 1.02; 95% CI 0.99-1.06)

**Hellman et al., 2025**	Sweden	CC	92	HPV16, 18, 31, 33, 35, 39, 45, 51, 52, 56, 58, 59, 66	ddPCR	Pretreatment	Binary with cut-off based on median 150 copies/3mL	PFS	Not specified	FIGO I-IV	No difference in PFS was observed between ctHPV DNA levels over the median level across the whole cohort (p = 0.331).	Tumor diameter (cm) – significant association (p = 0.004)
**Huffman et al., 2024**	USA	AC	25	HPV16, 18, 31, 33, 35	NavDx (ddPCR)	Pre-treatment, repeatedly during treatment	NA	-	Median 12.6 months	T1-4, N0-3, M0-1	NR	sum of longest tumor diameters of RECIST target lesions (Spearman's ρ = 0.72, *p* < 0.0001)
**Cao et al. 2012**	Japan	OPC	37	HPV16, 18	qPCR	Pre-treatment, during RT	NA	-	NA	Stage I-IVA (AJCC 6)	NR	MTV50-T (Pearson's r = 0.12, p = 0.47);<br>MTV50-N (Pearson's r = 0.62, p <0.001, Spearman's ρ = 0.53, p = 0.002)<br>MTV50-T+N (Pearson's r = 0.51, p = 0.001)
**Bønløkke et al., 2022**	Denmark	CC	60	HPV16, 18	ddPCR	Pre-treatment	NA	-	NA	FIGO IA1–IVA	NR	Tumor diameter (mm, linear regression r^2 = 0.25, p < 0.005);
**Hanna et al., 2019**	USA	OPC	21	HPV16, 18, 31, 33, 45	ddPCR	Follow-up	NA	-	Median 3,43 months	Incurable or metastatic disease	NR	TTB (Spearman's ρ = 0.79, p < 0.01)
**Lee et al., 2020**	United Kingdom	AC	21	HPV16, 18, 31, 33, 35, 45, 52, 58	NGS	Pre-treatment, 12 weeks post-CRT	NA	-	Median 16.8 months	Stage I-IIIB (AJCC 6)	NR	GTV-T+N (linear regression r^2 = 0.3, p = 0.13)
**Tatsumi et al., 2024**	Japan	OPC, HNCUP	55	HPV16	ddPCR	Pre-treatment	NA	-	NA	Stage I-IV (UICC 8)	NR	Whole body MTV (Spearman's ρ = 0.53, p < 0.0005);<br>largest lesion MTV – no significant correlation

**Lam et al., 2024**	USA	OPC	70	HPV16, 18, 31, 33, 35	NavDx (ddPCR)	Pre-treatment	NA	-	NA	T0-4, N0-2, M0 (AJCC 8)	NR		Tumor diameter (simple linear regression analysis – r = 0.02 [95% CI, −0.22 to 0.25]),<br>Size of metastatic lymph node deposit – (r = 0.19 [95% CI, −0.07 to 0.43])
**Taguchi et al., 2025**	Japan	CC	26	HPV16, 18, 52, 58	ddPCR	Pre-treatment; during treatment; after treatment up to 6 months	NA	-	6 months	FIGO I-IV	NR		Tumor diameter (mm, Pearsons' r=0.624, P<0.01)
**Huttinger et al., 2025**	USA	OPC	17	HPV16, 18, 31, 33, 35	NavDx (ddPCR)	Pretreatment; 2–3 weeks after IC; 3 months after CRT	NA	-	3 months	T1-4, N1-3	NR		GTV-T ($R^2 = 0.33$, p = 0.02),<br>GTV-N ($R^2 = 0.01$, p = 0.7),<br>Total disease burden (R2 = 0.02, p = 0.6)
**Lee et al., 2025**	USA	OPC	94	HPV16, 18, 31, 33, 35	NavDx (ddPCR)	Pre-treatment	NA	-	NA	T0-3, N0-3 (AJCC 7, 8)	NR		Largest metastatic lymph node dimension (multivariate linear regression r = 0.63, p < 0.001),<br>Total lymph node dimension (r = 0.41, p < 0.001);<br>Number of metastatic nodes (r = 0.52, p = 0.01);<br>Tumor diameter – no significant association
**Almerén et al., 2025**	Sweden	OPC, HNCUP	51	HPV16, 18, 33, 35, 56, 59	ddPCR	Pre-treatment, post-treatment, follow-up	NA	-	Median 1.35 months	T0-4, N0-3 (AJCC 8)	NR		GTV-T+N (Kendal's τ = 0.39, p < 0.01);<br>GTV-N (Kendal's τ = 0.26, p < 0.01);<br>GTV-T (Kendal's τ = 0.22, p < 0.05);<br>sum of diameter of target lesions (Kendal's τ = 0.29, p < 0.01);<br>tumor diameter, sum of diameter of target lymph nodes – no significant correlation
**Seo et al., 2025**	USA	CC	66	HPV16 18, 31, 33, 35, 39, 45, 51, 52, 56, 58, 59, 68	ddPCR	Pre-treatment, during treatment, (weeks 1, 3, and 5 of CRT),	NA	-	Median 23 months	FIGO I-IV	NR		Baseline cfDNA levels were not significantly correlated to GTV (p = 0.24) or SUVmax (p = 0.57).

	3-4 months post-treatment	

* – values calculated from raw data;

** – values calculated based on Kaplan-Meier curves;

*** – values calculated by combining 2 subgroup-specific HR estimates

[a] – cancer types included in survival and/or tumor burden analysis;

[b] – number of patients included in survival and/or tumor burden analysis;

[c] – all follow-up data reported in the included studies were converted to months to ensure comparability;

[d] – associations listed in this column are based on the initial (baseline or pre-treatment) ctHPV measurement reported in each study, regardless of additional measurements at later timepoints.

[e] – study-specific labels for nodal volume, primary tumor volume, and total (primary + nodal) gross tumor volume were considered synonymous with GTV-N, GTV-T, and GTV-T+N, respectively.

AC – anal cancer, AJCC – American Joint Committee on Cancer, aOR – adjusted odds ratio, CC – cervical cancer, CHT – chemotherapy, CRT – chemoradiotherapy, ctHPV – circulating tumor HPV DNA, dPCR – digital polymerase chain reaction, ddPCR — droplet digital polymerase chain reaction, DFS – disease-free survival, DSS – disease-specific survival, FFP – freedom from progression, FIGO – International Federation of Gynecology and Obstetrics, GTV-N – gross nodal tumor volume, GTV-T – gross primary tumor volume, GTV-T+N – total gross tumor volume (sum of GTV-T and GTV-N), HNSCC – head and neck squamous cell carcinoma, HPV – human papillomavirus, HR – hazard ratio, IC – induction chemotherapy, LRFS – locoregional recurrence-free survival, MFS – metastasis-free survival, MTV – metabolic tumor volume, MTV50-N – nodal MTV at 50% SUVmax, MTV50-T – primary-tumor MTV at 50% SUVmax, MTV50-T+N – primary + nodal MTV at 50% SUVmax, NA – not applicable, NavDx – commercial ddPCR assay for circulating tumor-tissue modified viral human papillomavirus DNA, NGS – next-generation sequencing, NR – not reported, OPC – oropharyngeal cancer, OS – overall survival, PFS – progression-free survival, qPCR – quantitative real-time PCR, R^2 – coefficient of determination, RECIST – Response Evaluation Criteria in Solid Tumors, RFS – recurrence-free survival, ROC – receiver operating characteristic, RPFS – recurrence/persistence-free survival, RT – radiotherapy, SCCUP – squamous cell carcinoma of unknown primary, SUVmax – standardized uptake value maximum, UICC – Union for International Cancer Control, TTB - total tumor burden (sum of the largest diameter of all detectable lesions on imaging)
